# Vitamin D Deficiency and Associated Factors in Children: A Multicenter Study of 21,811 Samples in Southern China

**DOI:** 10.3389/ijph.2024.1607411

**Published:** 2025-01-06

**Authors:** Chuican Huang, Sijia Liu, Chen Cheng, Shiyun Chen, Qing Luo, Yan Huang, Yanxian Yao, Xixia Ye, Haizhen Wang, Liangyi Luo, Junwei Xie, Hongai Li, Yumei Duan, Beibei Liu, Wenting Cao, Fangfang Zeng, Wei Xiang, Lichun Fan

**Affiliations:** ^1^ Department of Child Healthcare, Hainan Women and Children’s Medical Center (Children’s Hospital Affiliated to Hainan Medical University), Haikou, China; ^2^ Department of Public Health and Preventive Medicine, School of Medicine, Jinan University, Guangzhou, China; ^3^ Department of Epidemiology, International School of Public Health and One Health, Hainan Medical University, Haikou, China; ^4^ National Health Commission (NHC) Key Laboratory of Control of Tropical Diseases, Hainan Medical University, Haikou, China

**Keywords:** children, vitamin D deficiency, risk factors, cross-sectional study, Southern China

## Abstract

**Objective:**

To investigate the prevalence of vitamin D deficiency (VDD) in children/adolescents in extreme southern China.

**Methods:**

This multicenter, cross-sectional study included 21,811 children aged 0–18 years from 18 districts in Hainan Province, using a multistage stratified random sampling method from January 2021 to March 2022.

**Results:**

Serum 25(OH)D levels decreased with age (p trend <0.001). VDD prevalence increased significantly from 3.7% (95% CI: 3.2, 4.3) in children aged 0–3 years to 43.5% (95% CI: 42.1, 45.0) in those aged 13–18 years. Girls and urban residents showed higher deficiency rates. Adolescents (13–18 years) had the highest prevalence of VDD (43.5%), while toddlers (0–3 years) had the lowest (3.7%). Factors influencing vitamin D status included gender, urban residency, and breastfeeding duration. Seasonal variations showed higher deficiency rates in autumn, particularly among preschoolers. Regional differences were noted, with the highest deficiency in semiarid and subhumid zones for various age groups.

**Conclusion:**

A significant increase in VDD with age, particularly among adolescents, urban girls, and during autumn, emphasizing the need for targeted strategies.

## Introduction

Vitamin D is a crucial fat-soluble vitamin essential for children’s growth and long-term health. It aids in calcium and phosphorus metabolism, bone development, and various physiological functions, including immune response and cellular differentiation [1, 2]. Recently, vitamin D deficiency (VDD) has become a significant global health issue, contributing to diseases in children and adolescents such as dental caries, asthma, delayed growth, and rickets, while also increasing the risk of hip fractures and overall mortality later in life, thereby severely impacting quality of life [3–5].

Globally, the issue of VDD received widespread attention. Although regions with ample sunlight are typically considered to have a lower risk of VDD, studies show that the prevalence among children is still alarmingly high [6, 7]. Additionally, the VDD prevalence varies by geographic region, with about 15% of U.S. children aged 1–11 years [8] and 25.44% among children aged 6–18 years in India [9]. VDD is particularly common in countries where foods lack vitamin D fortification, such as Germany (45.6%), Australia (31%), Canada (61%), and Iran (81%) [4, 10, 11]. In China, a national cross-sectional study involving 10,696 children and teenagers aged 6–18 years from northern cities (Beijing, Changchun, Jinan, and Yinchuan) and southern cities (Shanghai, Chongqing, and Chengdu) found that 80% had VDD (25[OH]D < 50 nmol/L) [12]. A nationwide study reported VDD in 8.9% of 1,435 children aged 3–5 years [13].

The epidemiology of VDD is affected by various factors, including dark skin, southern latitude, limited sunlight exposure, impaired vitamin D metabolism, maternal VDD during pregnancy [14], and prolonged breastfeeding without supplementation [15]. Dietary habits, sunlight exposure, and socioeconomic factors related to physical activity are key contributors to VDD in children [16, 17]. Notably, VDD is prevalent among obese individuals [18], who may have lower dietary vitamin D intake and reduced sunlight exposure. Research suggests that adipose tissue acts as a primary storage site for vitamin D, leading to decreased bioavailability due to sequestration in excess fat [19]. Sunlight exposure and breast milk are primary natural sources of vitamin D for newborns [20]. Unfortunately, breast milk does not provide sufficient vitamin D, prompting experts to recommend supplementation within the first week of life to prevent rickets [21]. VDD has also been observed in sun-rich Asian countries like Malaysia, Indonesia, Thailand, and Vietnam, as well as in tropical Colombia [22]. Large-scale studies are needed to further investigate VDD status in tropical regions with high ultraviolet B (UV-B) radiation. Implementing targeted preventive and intervention strategies for VDD in these populations is essential.

Hainan Province, the largest tropical island in the South China Sea, is distinct due to its tropical monsoonal climate, geographical features, and diverse ethnicities [23]. However, there is limited data on vitamin D status and VDD prevalence and its related factirs in the region. To address this knowledge gap, we analyzed data across a broader age range to assess vitamin D status and VDD with strata of factors such as age and seasons in children in southern China.

## Methods

### Procedures

This research is a cross-sectional study using a province-wide representative sample of children in Hainan, aged 0–18 years (mean age: 8.00 ± 4.81 years). Conducted from January 2021 to March 2022, the study employed a multistage stratified random sampling method. Districts and communes were categorized by economic development, and one district or township was randomly selected from each category. Then, 1-2 streets or administrative villages were chosen within these locations. The detection rate and details of the 18 cities and counties are provided in the Supplementary Material (Supplementary Tables S1–S3; Supplementary Figures S1, S2). Hainan Province is divided into five regions based on climate: east, west, north, south, and central. The eastern part is classified as the humid zone, which includes Wenchang, Qionghai, and Wanning. The northern region is classified as the semihumid zone, encompassing Haikou, Ding’an County, Danzhou, Lingao County, Chengmai County, and part of Tunchang County. The southern region represents the semiarid and semihumid zone, which includes Sanya and three autonomous counties: Lingshui, Ledong, and Baoting. The western region is identified as the semiarid zone, comprising Dongfang and Changjiang autonomous counties. In contrast, the central region is characterized as the mountainous humid zone, which includes Wuzhishan and two autonomous counties: Qiongzhong and Baisha.

This study followed the Declaration of Helsinki guidelines and received approval from the Hainan Women and Children’s Medical Center Ethics Committee (No. 2021[005]), with written informed consent obtained from parents or guardians of all participants.

### Participants

All potential participants in the sampled locations were invited to join our survey. Inclusion criteria were children and adolescents who: i) have resided in urban or rural areas for an extended period and are registered there, or ii) have relocated but lived in these areas for more than two-thirds of their age. Exclusion criteria included those who: i) have relocated but lived in the selected locations for less than two-thirds of their age; ii) have specific diseases, including severe chronic conditions (e.g., heart disease, chronic nephritis, chronic bronchitis, asthma, endocrine disorders, and neurological diseases), infectious diseases (e.g., tuberculosis and hepatitis), endemic diseases, moderate or severe rickets, and limb disabilities; iii) are less than 1 month post-recovery from acute diseases (e.g., pneumonia and dysentery); iv) have had a fever lasting over 7 days in the 2 weeks prior; or v) have experienced diarrhea exceeding five times a day for 5 days or more.

### Data Collection

In each city, survey teams were established based on local conditions, comprising 10 to 13 professionals. Two pediatricians with relevant clinical experience reviewed physical examination data and diagnosed diseases, while the other team members were skilled health workers. Trained physicians conducted face-to-face interviews with eligible children or their guardians using structured questionnaires tailored for two age groups: under 3 years and 3–18 years. Both questionnaires collected data on household socioeconomic status and feeding practices. Before data entry, data were checked and errors corrected by a team of data management unit member, city level responsible personnel, and principal investigator. All field workers were trained and certified in their data collection techniques before they were permitted to collect data in field. Data abstraction and double entry verification were performed by specifically trained data entry staff in compliance with Good Clinical Practice guidelines.

### Specimen Collection and 25(OH)D Measurements

Parents and children were instructed to fast overnight (8–10 h) before sample collection, except for infants. Fasting venous blood was drawn into two plasma anticoagulant tubes containing heparin or EDTA. Samples were transported at 2°C–8°C to nearby collection centers within 24 h. After labeling and recording, they were sent via cold chain to the central laboratory at Hainan Maternal and Child Health Hospitals. Serum was separated and aliquoted within 6 h, centrifuged at 13,000 rpm for 10 min at 4°C, and stored at −20°C until analysis. The serum concentration of 25(OH)D was measured using liquid chromatography-mass spectrometry (LC-MS/MS, API 3200, AB Sciex Pte, United States) with a limit of detection (LOD) of 4 ng/mL. The serum samples underwent separation using liquid chromatography, during which a chromatographic column was employed to remove interfering substances. This was followed by mass spectrometry using a high-resolution mass spectrometer to quantify serum 25(OH)D concentrations based on mass spectrum interpretation. Concentrations of 25(OH)D2 and 25(OH)D3 were summed to calculate the total 25(OH)D concentration. To ensure accuracy and reproducibility, we included internal duplicate quality control samples during the assay. These internal controls demonstrated coefficients of variation (CVs) of 3.5% for 25(OH)D2 and 1.2% for 25(OH)D3. Additionally, data analysis was consistently calibrated against a standard curve. Vitamin D deficiency (VDD) was defined as a total 25(OH)D concentration [sum of 25(OH)D2 and 25(OH)D3] below 20 ng/mL (50 nmol/L) [24].

### Statistical Analysis

Baseline characteristics are presented as means and standard deviations (SD) or medians and interquartile ranges; categorical variables are reported as counts and proportions. Participants were divided into four age groups (0–3, 4–6, 7–12, and 13–18 years) primarily based on their educational stages: 0–3 years for preschool, 4–6 years for kindergarten, 7–12 years for primary school, and 13–18 years for secondary school. We used multiple imputation to impute the missing covariates. Additional demographic characteristics considered for further categorization included sex, nationality, region, BMI, vitamin D supplementation during pregnancy, premature birth, conception and delivery methods, number of births, birth weight, exclusive breastfeeding for 6 months or more, growth assessments, and family income levels. Serum levels of 25(OH)D2, 25(OH)D3, and total 25(OH)D were measured across the age groups, with differences analyzed using the chi-square test. The overall and stratified prevalence of VDD, including 95% confidence intervals, case numbers, and sample sizes, were evaluated in each age group.

We calculated the demographic covariates and climate-specific stratified distributions of 25(OH)D2, 25(OH)D3, and total 25(OH)D levels, along with the stratified prevalence (95% CIs) of VDD, including case numbers and sample sizes across age groups to assess distribution and potential differentials. Mean serum 25(OH)D values were analyzed by age-sex, age-region, and age-feeding method using box plots and the Mann-Whitney-Wilcoxon test. Line charts and forest plots were created to visually compare the prevalence of VDD by age-sex, age-region, and region. We identified gender, nationality, region, BMI, prenatal vitamin D supplementation, preterm birth, conception and delivery methods, number of births, birth weight, duration of exclusive breastfeeding, growth assessments, and family income as covariates influencing the prevalence of VDD (dependent variable) through multivariable logistic regression models.

All analyses were adjusted with a weighting factor to address imbalances from frame and nonresponse issues. Statistical analyses were performed using R software version 4.0.2 (R Core Team, 2020). A two-sided P-value of 0.05 was deemed statistically significant.

## Results

### Basic Characteristics of the Study Population

The analysis utilized a sample of 21,811 children and adolescents from Hainan Province, China, encompassing 18 cities and counties. The age distribution of the sample included 4,382 participants aged 0–3 years, 4,734 aged 4–6 years, 8,177 aged 7–12 years, and 4,518 aged 13–18 years. The basic characteristics of the study population are summarized in Table 1. The study population were evenly split between men and women, mostly Han Chinese, urban residents, non-premature, naturally conceived and born, single children, weighing 2.5–4.0 kg at birth, with minimal vitamin D supplementation during pregnancy, exclusively breastfed for over 6 months, assessed as growing normally, and from low-income families.

**TABLE 1:** Clinical characteristics of study population (China, 2022).

Covariates	Percentages (95% CIs)^a^			
	0–3 years	4–6 years	7–12 years	13–18 years
Gender				
Male	54.8 (53.4–56.3)	51.9 (50.5–53.3)	49.6 (48.6–50.7)	48.3 (46.8–49.8)
Female	45.2 (43.7–46.6)	48.1 (46.7–49.5)	50.4 (49.3–51.4)	51.7 (50.2–53.2)
Nationalities				
Han	76.4 (75.1–77.7)	82.6 (81.4–83.7)	83.3 (82.2–84.4)	80.3 (78.8–81.8)
Li	21.0 (19.7–22.2)	15.1 (14.0–16.2)	15.2 (14.1–16.2)	16.1 (14.7–17.5)
Others	2.6 (2.2–3.1)	2.3 (1.9–2.8)	1.5 (1.2–1.9)	3.6 (2.9–4.3)
Regions				
Urban	34.6 (33.1, 36.0)	55.4 (54.0, 56.8)	74.6 (73.7, 75.6)	77.8 (76.6, 79.1)
Rural	64.4 (62.9, 65.8)	43.4 (42.0, 44.8)	25.4 (24.4, 26.3)	22.1 (20.9, 23.3)
Quartile of BMI (kg/m^2^)				
Q1	25.1 (24.6, 26.2)	50.2 (49.6, 51.4)	27.5 (27.0, 28.6)	1.5 (1.3, 1.7)
Q2	40.7 (40.1, 41.9)	32.6 (32.0, 33.7)	25.5 (25.0, 26.6)	6.9 (6.6, 7.5)
Q3	30.2 (29.6, 31.3)	12.9 (12.4, 13.7)	23.7 (23.1, 24.7)	31.4 (30.8, 32.5)
Q4	3.9 (3.7, 4.4)	4.3 (4.1, 4.8)	23.2 (22.7, 24.2)	60.2 (59.5, 61.4)
Vitamin D supplement during gestation				
Never	75.3 (74.7, 76.3)	64.9 (64.3, 66.1)	46.2 (45.6, 47.4)	41.8 (41.2, 43.0)
Seldom	8.7 (8.3, 9.4)	25.7 (25.1, 26.7)	44.8 (44.2, 46.0)	46.7 (46.0, 47.8)
Often	4.7 (4.4, 5.2)	4.8 (4.5, 5.3)	6.9 (6.6, 7.5)	9.3 (8.9, 10.0)
Always	11.3 (10.9, 12.1)	4.6 (4.3, 5.1)	2.1 (1.9, 2.4)	2.2 (2.0, 2.6)
Premature birth ^b^				
Yes	7.8 (7.0–8.7)	8.8 (7.8–9.7)	9.7 (8.8–10.7)	15.7 (14.2–17.2)
No	92.2 (91.3–93.0)	91.2 (90.3–92.2)	90.3 (89.3–91.2)	84.3 (82.8–85.8)
Conception ways				
Natural pregnancy	97.9 (97.5–98.3)	97.9 (97.4–98.3)	98.4 (98.1–98.8)	98.9 (98.5–99.3)
Assisted reproduction	2.1 (1.7–2.5)	2.1 (1.7–2.6)	1.6 (1.2–1.9)	1.1 (0.7–1.5)
Delivery methods				
Natural Childbirth	71.0 (69.6–72.4)	71.3 (69.9–72.7)	73.3 (72.0–74.6)	82.1 (80.7–83.6)
Cesarean section	29.0 (27.6–30.4)	28.7 (27.3–30.1)	26.7 (25.4–28.0)	17.9 (16.4–19.3)
Number of births				
Single birth	95.6 (95.0–96.2)	93.6 (92.8–94.3)	94.6 (93.9–95.2)	94.8 (93.9–95.6)
Multiple births	4.4 (3.8–5.0)	6.4 (5.7–7.2)	5.4 (4.8–6.1)	5.2 (4.4–6.1)
Birth weight (kg)				
<2.5	7.1 (6.3–7.8)	5.5 (4.8–6.2)	5.3 (4.6–6.0)	6.4 (5.4–7.4)
2.5–4.0	87.3 (86.3–88.3)	82.7 (81.5–83.9)	80.4 (79.2–81.6)	73.9 (72.2–75.7)
>4.0	5.6 (4.9–6.3)	11.8 (10.8–12.8)	14.3 (13.2–15.3)	19.7 (18.1–21.3)
Exclusively breastfed for 6 months or more				
Yes	56.7 (55.2–58.2)	79.8 (78.5–81.0)	86.1 (85.1–87.1)	92.6 (91.6–93.6)
No	43.3 (41.8–44.8)	20.2 (19.0–21.5)	13.9 (12.9–14.9)	7.4 (6.4–8.4)
Growth and development assessment				
Retardation	15.6 (14.4–16.7)	14.7 (13.6–15.7)	7.5 (6.9–8.1)	7.4 (6.7–8.2)
Normal	80.1 (78.8–81.4)	82.0 (80.8–83.2)	87.6 (86.9–88.4)	87.6 (86.6–88.6)
Obesity	4.3 (3.7–5.0)	3.3 (2.8–3.9)	4.8 (4.4–5.3)	4.9 (4.3–5.6)
Family annual income (yuan)				
0–50,000	75.0 (73.6–76.3)	63.8 (62.3–65.4)	68.0 (66.7–69.4)	69.8 (68.1–71.6)
50,000–100,000	15.8 (14.7–17.0)	20.2 (19.0–21.5)	19.0 (17.8–20.1)	19.1 (17.6–20.6)
>100,000	9.2 (8.3–10.1)	15.9 (14.8–17.1)	13.0 (12.0–14.0)	11.1 (9.9–12.3)

Abbreviations: 95%CIs, 95% confident intervals.

Note:.

^a^ Percentage (95%CIs) of all testing sample.

^b^ Preterm birth referred to a child born at a gestational age greater than 28 weeks and less than 36 weeks.

### Circulating 25(OH)D2, 25(OH)D3, and 25(OH)D Levels

Table 2 shows the distribution of plasma vitamin D concentrations (ng/mL) across age groups. Adolescents aged 13–18 years had lower 25(OH)D levels, with a median of 20.86 ng/mL, compared to 35.99 ng/mL for ages 0–3 years, 26.50 ng/mL for ages 4–6 years, and 23.42 ng/mL for ages 7–12 years. Similar trends were observed for 25(OH)D2 and 25(OH)D3, indicating vitamin D levels decreased significantly with age, correlating with a higher prevalence of deficiency among older adolescents.

**TABLE 2:** Stratified levels of 25-hydroxyvitamin D in children and adolescence across age groups (China, 2022).

Covariates	0–3 years		4–6 years		7–12 years		13–18 years	
	25(OH)D^a^	*P*	25(OH)D^a^	*P*	25(OH)D^a^	*P*	25(OH)D^a^	*P*
Overall	35.99 (29.62, 43.77)		26.50 (22.58, 30.81)		23.42 (19.78, 27.56)		20.86 (17.14, 24.56)	
Gender		**<0.001**		**<0.001**		**<0.001**		**<0.001**
Male	36.64 (30.34, 44.41)		27.17 (23.18, 31.70)		24.66 (20.90, 28.66)		21.91 (18.35, 25.91)	
Female	35.24 (28.67, 42.92)		25.90 (22.08, 30.03)		22.36 (18.78, 26.32)		19.84 (16.20, 23.40)	
Nationalities		0.159		<0.001		0.920		0.220
Han	36.06 (29.44, 44.02)		26.54 (22.46, 30.90)		23.52 (19.90, 27.62)		21.10 (17.24, 24.56)	
Li	36.67 (30.82, 44.25)		27.50 (24.11, 32.01)		23.82 (19.85, 27.71)		21.21 (17.74, 25.10)	
Others	35.75 (28.84, 44.48)		26.24 (22.92, 31.59)		23.62 (19.32, 28.31)		21.50 (18.28, 25.45)	
Regions		**<0.001**		**<0.001**		**<0.001**		**<0.001**
Urban	33.88 (27.70, 41.79)		25.20 (21.18, 29.45)		23.04 (19.38, 27.06)		21.10 (17.26, 24.84)	
Rural	37.14 (30.67, 44.66)		28.00 (24.36, 32.44)		24.76 (20.90, 28.95)		20.02 (16.98, 23.46)	
Quartile of BMI (kg/m^2^)		**<0.001**		**0.013**		**<0.001**		0.360
Q1	30.98 (26.82, 36.52)		27.22 (23.18, 31.14)		24.80 (21.24, 28.72)		20.88 (18.58, 26.10)	
Q2	31.78 (26.16, 37.74)		26.62 (23.02, 30.92)		23.88 (20.06, 28.06)		21.50 (16.42, 24.34)	
Q3	33.58 (26.96, 41.22)		25.09 (22.04, 29.66)		22.79 (19.25, 26.66)		20.76 (17.25, 24.18)	
Q4	33.68 (24.66, 40.36)		25.01 (20.85, 31.28)		22.78 (19.27, 26.26)		21.42 (17.80, 25.04)	
Vitamin D supplement during gestation		**<0.001**		**<0.001**		0.853		0.243
Never	31.84 (27.24, 38.46)		26.84 (23.14, 31.21)		23.42 (19.84, 27.45)		21.18 (17.31, 24.51)	
Seldom	28.48 (24.35, 33.73)		25.54 (22.06, 29.78)		23.76 (20.18, 27.65)		21.28 (17.59, 24.98)	
Often	33.64 (27.20, 39.44)		27.00 (23.88, 30.78)		23.71 (19.92, 27.87)		21.42 (18.30, 24.68)	
Always	37.72 (31.36, 44.37)		29.90 (27.65, 35.67)		23.13 (19.35, 27.87)		21.76 (18.30, 25.78)	
Premature birth ^b^		0.700		0.740		0.772		0.225
Yes	36.12 (30.24, 41.80)		26.72 (23.59, 31.80)		23.56 (19.91, 27.93)		20.32 (17.00, 24.22)	
No	36.51 (30.14, 44.54)		26.58 (22.50, 30.85)		23.60 (19.80, 27.78)		21.06 (17.34, 24.64)	
Conception ways		**0.022**		0.722		0.996		0.562
Natural pregnancy	36.24 (29.97, 44.17)		26.75 (22.78, 31.10)		23.58 (19.88, 27.64)		21.13 (17.36, 24.74)	
Assisted reproduction	33.76 (27.74, 40.53)		26.50 (22.25, 30.15)		23.56 (19.62, 28.84)		21.18 (15.71, 24.71)	
Delivery methods		0.480		0.160		0.152		0.546
Natural Childbirth	36.14 (29.89, 44.30)		26.80 (22.90, 31.09)		23.50 (19.86, 27.60)		21.20 (17.50, 24.75)	
Cesarean section	36.09 (30.10, 43.72)		26.44 (22.21, 31.03)		23.78 (19.97, 27.93)		20.94 (16.80, 24.78)	
Number of births		**<0.001**		0.779		0.804		0.880
Single birth	36.49 (30.16, 44.31)		26.68 (22.70, 31.05)		23.59 (19.90, 27.66)		21.12 (17.40, 24.76)	
Multiple births	31.93 (25.83, 38.67)		26.90 (23.14, 31.12)		23.38 (19.48, 27.60)		21.69 (16.46, 24.53)	
Birth weight (kg)		**<0.001**		**<0.001**		0.493		0.864
<2.5	37.84 (31.81, 45.07)		27.68 (24.18, 33.00)		23.86 (20.40, 28.72)		21.24 (17.63, 24.90)	
2.5–4.0	36.65 (30.26, 44.50)		26.48 (22.46, 30.78)		23.66 (19.83, 27.69)		21.24 (17.47, 24.64)	
>4.0	31.20 (26.11, 37.62)		27.44 (23.60, 32.03)		23.46 (19.91, 27.62)		20.82 (16.98, 24.97)	
Exclusively breastfed for 6 months or more		**<0.001**		**<0.001**		0.979		0.975
Yes	34.41 (28.26, 42.10)		26.56 (22.60, 30.86)		23.58 (19.85, 27.66)		21.16 (17.36, 24.76)	
No	38.54 (32.20, 46.25)		27.22 (23.26, 32.14)		23.46 (19.98, 27.57)		20.96 (17.53, 24.42)	
Growth and development assessment		0.116		**<0.001**		**<0.001**		0.277
Retardation	37.22 (31.21, 45.00)		27.12 (23.59, 31.13)		24.92 (21.17, 28.91)		20.60 (16.00, 24.96)	
Normal	36.77 (30.42, 44.64)		26.32 (22.32, 30.65)		23.26 (19.62, 27.38)		20.98 (17.26, 24.59)	
Obesity	36.28 (29.73, 46.07)		23.86 (20.75, 28.59)		23.26 (18.99, 27.10)		20.58 (17.09, 23.92)	
Family annual income (yuan)		**<0.001**		**<0.001**		0.308		0.559
0–50,000	37.12 (30.68, 44.99)		27.10 (23.42, 31.54)		23.63 (19.97, 27.72)		21.12 (17.31, 24.70)	
50,000–100,000	35.41 (29.62, 42.54)		26.02 (21.83, 30.47)		23.58 (20.03, 27.46)		21.33 (17.82, 25.00)	
>100,000	31.55 (26.47, 40.50)		25.63 (21.20, 29.97)		23.34 (19.36, 27.38)		20.93 (17.04, 25.03)	

Note: Levels of 25(OH)D2(ng/mL), 25(OH)D3(ng/mL), and 25(OH)D(ng/mL) were expressed as median (P25, P75).

^a^ 25(OH)D = 25(OH)D2+25(OH)D3.

^b^ Preterm birth referred to a child born at a gestational age greater than 28 weeks and less than 36 weeks.

**FIGURE 1:**
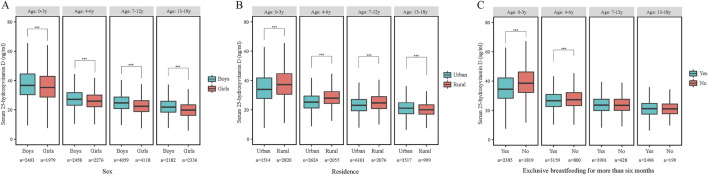
The serum 25-hydroxyvitamin D concentrations by age and sex **(A)**, residence **(B)** and excessively breastfed **(C)** (China, 2022). Abbreviations: ***p < 0.001, Mann-Whitney-Wilcoxon test.

Regarding stratified levels, girls were more likely than boys to have lower serum levels of 25(OH)D2, 25(OH)D3, and 25(OH)D as they aged (P < 0.001) (Figure 1A). Low vitamin D levels, except for 25(OH)D2, were more common in urban children (P < 0.001) (Figure 1B), those conceived via artificial insemination, with low BMI, weighing over 4.0 kg at birth, and diagnosed with obesity. Low vitamin D was also linked to minimal prenatal vitamin D supplementation and higher household income. Among children under 6 of other nationalities, exclusive breastfeeding for 6+ months was associated with lower vitamin D levels (P < 0.001), but this trend reversed for those aged 7 and above (P > 0.05) (Figure 1C) (Table 2; Supplementary Table S4).

### Climate-Specific Zones Stratified Circulating Vitamin D Concentrations

As shown in Table 3; Supplementary Table S5, regional variations in low vitamin D status were observed across age groups. Among toddlers (0–3 years) and teenagers (13–18 years), the highest 25(OH)D3 and 25(OH)D levels were found in the mountainous humid zone, while the lowest were in the humid and subhumid zones, respectively. For preschoolers (4–6 years), the highest levels were in the humid zone and the lowest in the subhumid zone. In middle childhood (7–12 years), both 25(OH)D3 and 25(OH)D peaked in the semiarid zone and were lowest in the mountainous humid zone (P < 0.001).

**TABLE 3:** Climate-specific zones stratified levels of serum 25-hydroxyvitamin D across age groups (China, 2022).

Climate-specific zones	0–3 years		4–6 years		7–12 years		13–18 years	
	25(OH)D^a^	*P*	25(OH)D^a^	*P*	25(OH)D^a^	*P*	25(OH)D^a^	*P*
		<0.001		<0.001		<0.001		<0.001
Humid zone	31.33 (26.63, 37.42)		29.30 (25.20, 33.73)		23.42 (19.73, 27.81)		21.40 (18.63, 24.76)	
Sub-humid zone	35.74 (28.53, 44.50)		25.42 (21.31, 29.78)		23.08 (19.40, 27.14)		20.68 (16.70, 24.07)	
Mountainous humid zone	38.14 (31.82, 45.91)		26.28 (22.94, 30.00)		22.28 (18.65, 26.44)		21.94 (17.72, 26.40)	
Semi-arid and sub-humid zone	35.66 (29.96, 44.02)		27.20 (23.80, 31.16)		24.74 (21.20, 28.86)		21.07 (17.48, 25.28)	
Semi-arid zone	32.06 (27.66, 39.69)		27.26 (24.13, 31.53)		29.52 (23.30, 31.80)		21.28 (18.20, 24.54)	

Note: Levels of 25(OH)D(ng/mL) was expressed as median (P25, P75).

^a^ 25(OH)D = 25(OH)D2+25(OH)D3.

### Prevalence of VDD

Table 4 shows the prevalence of 25(OH)D deficiency by age. The highest prevalence was among teenagers aged 13–18 years (43.5%), followed by children aged 7–12 years (26.2%) and preschoolers aged 4–6 years (13.2%), with the lowest prevalence in toddlers aged 0–3 years (3.7%).

**TABLE 4:** Stratified prevalence of 25-hydroxyvitamin D deficiency in children and adolescence across age groups (China, 2022).

Covariates	0–3 years			4–6 years			7–12 years			13–18 years		
	Prevalence (%) (95% CI)	Testing sample	*P*	Prevalence (%) (95% CI)	Testing sample	*P*	Prevalence (%) (95% CI)	Testing sample	*P*	Prevalence (%) (95% CI)	Testing sample	*P*
Overall	3.7 (3.2, 4.3)	4,382		13.2 (12.3, 14.2)	4,734		26.2 (25.3, 27.2)	8,177		43.5 (42.1, 45.0)	4,518	
Gender			**0.014**			**0.001**			**<0.001**			**<0.001**
Male	3.1 (2.4, 3.8)	2,403		11.7 (10.4, 13.0)	2,458		19.6 (18.4, 20.8)	4,059		35.1 (33.1, 37.1)	2,182	
Female	4.5 (3.6, 5.4)	1979		14.9 (13.4, 16.3)	2,276		32.7 (31.3, 34.2)	4,118		51.4 (49.4, 53.4)	2,336	
Nationalities			0.157			**<0.001**			**<0.001**			0.761
Han	3.8 (3.1, 4.5)	3,215		14.0 (12.9, 15.2)	3,268		25.6 (24.2, 27.0)	3,775		41.8 (39.7, 43.8)	2,172	
Li	2.5 (1.5, 3.5)	883		7.5 (5.4, 9.6)	598		25.4 (22.2, 28.7)	688		41.1 (36.4, 45.7)	436	
Others	2.7 (0.9, 7.6)	111		12.0 (5.3, 18.6)	92		30.0 (19.3, 40.7)	70		38.1 (28.5, 47.8)	97	
Regions			**<0.001**			**<0.001**						**<0.001**
Urban	7.0 (5.7, 8.3)	1,514		19.3 (17.8, 20.8)	2,624		28.5 (27.3, 29.6)	6,101		41.7 (40.1, 43.3)	3,517	
Rural	2.0 (1.5, 2.5)	2,820		5.6 (4.6, 6.6)	2055		19.7 (17.9, 21.4)	2076		49.8 (46.7, 53.0)	999	
Quartile of BMI (kg/m^2^)			0.127			0.059			**<0.001**			**0.037**
Q1	3.3 (2.1, 4.5)	878		14.6 (12.8, 16.3)	1,572		20.0 (18.3, 21.8)	2,100		47.6 (35.3, 60.0)	63	
Q2	2.2 (1.4, 2.9)	1,424		17.6 (15.3, 20.0)	1,020		25.9 (23.9, 27.8)	1948		44.8 (39.2, 50.5)	299	
Q3	2.6 (1.7, 3.6)	1,057		16.1 (12.5, 19.7)	403		29.3 (27.2, 31.4)	1807		46.0 (43.3, 48.6)	1,357	
Q4	5.1 (1.4, 8.8)	137		21.5 (14.6, 28.4)	135		31.8 (29.7, 34.0)	1771		41.4 (39.5, 43.3)	2,598	
Vitamin D supplement during gestation			**<0.001**			**<0.001**			0.794			0.451
Never	2.2 (1.7, 2.8)	2,735		13.7 (12.2, 15.3)	1797		26.2 (24.4, 28.1)	2088		42.3 (39.4, 45.1)	1,131	
Seldom	7.9 (4.9, 10.9)	316		21.4 (18.4, 24.4)	710		25.1 (23.2, 26.9)	2027		41.9 (39.1, 44.6)	1,261	
Often	7.1 (3.2, 11.0)	169		24.1 (16.8, 31.3)	133		25.3 (20.5, 30.1)	312		37.1 (31.1, 43.0)	251	
Always	2.4 (0.9, 3.9)	412		15.7 (9.4, 22.1)	127		28.0 (18.8, 37.1)	93		38.3 (26.0, 50.6)	60	
Premature birth ^a^			0.765			**0.037**			0.856			**0.014**
Yes	3.7 (1.7, 5.8)	321		9.7 (6.5, 13.0)	318		25.5 (21.1, 29.9)	376		48.7 (43.5, 53.9)	355	
No	3.4 (2.8, 4.0)	3,771		13.9 (12.8, 15.1)	3,309		26.0 (24.5, 27.4)	3,482		41.7 (39.5, 43.9)	1905	
Conception ways			0.224			0.736			0.624			0.589
Natural pregnancy	3.5 (3.0, 4.1)	4,123		13.0 (12.0, 14.1)	3,882		25.6 (24.3, 26.9)	4,463		41.6 (39.7, 43.4)	2,676	
Assisted reproduction	1.1 (0.2, 6.2)	88		11.8 (4.9, 18.6)	85		28.2 (17.7, 38.6)	71		36.7 (19.4, 53.9)	30	
Delivery methods			0.620			**0.001**			0.647			0.535
Natural Childbirth	3.4 (2.7, 4.0)	2,978		12.0 (10.8, 13.2)	2,797		25.7 (24.2, 27.1)	3,294		41.0 (39.0, 43.1)	2,169	
Cesarean section	3.7 (2.6, 4.8)	1,216		15.8 (13.7, 18.0)	1,125		25.0 (22.5, 27.4)	1,201		42.6 (38.1, 47.0)	472	
Number of births			**0.006**			0.838			0.488			0.990
Single birth	3.3 (2.7, 3.9)	3,995		13.2 (12.1, 14.3)	3,644		25.5 (24.2, 26.9)	4,286		41.5 (39.6, 43.4)	2,564	
Multiple births	7.1 (3.4, 10.8)	184		12.7 (8.6, 16.9)	251		27.5 (22.0, 33.1)	247		41.5 (33.4, 49.7)	142	
Birth weight (kg)			**<0.001**			**0.003**			0.516			0.551
<2.5	4.5 (2.1, 6.9)	290		9.4 (5.4, 13.4)	203		22.3 (16.7, 27.9)	211		38.8 (31.1, 46.6)	152	
2.5–4.0	2.8 (2.3, 3.4)	3,590		14.1 (12.8, 15.3)	3,054		25.8 (24.3, 27.4)	3,181		40.8 (38.5, 43.1)	1753	
>4.0	8.6 (5.0, 12.2)	232		8.9 (6.3, 11.6)	436		25.5 (21.9, 29.1)	564		43.1 (38.6, 47.6)	466	
Exclusively breastfed for 6 months or more			**<0.001**			**<0.001**			0.748			0.496
Yes	5.1 (4.2, 6.0)	2,385		13.9 (12.7, 15.1)	3,159		25.8 (24.4, 27.1)	3,901		41.7 (39.7, 43.6)	2,496	
No	1.4 (0.9, 2.0)	1819		9.4 (7.4, 11.4)	800		25.2 (21.8, 28.6)	628		39.2 (32.4, 46.0)	199	
Growth and development assessment			**0.009**			**<0.001**			**<0.001**			0.361
Retardation	2.5 (1.3, 3.8)	596		9.5 (7.2, 11.9)	609		18.2 (15.0, 21.3)	577		47.1 (41.6, 52.5)	323	
Normal	3.2 (2.6, 3.8)	3,062		14.3 (13.1, 15.5)	3,404		27.1 (26.0, 28.1)	6,728		43.0 (41.4, 44.5)	3,801	
Obesity	7.3 (3.3, 11.2)	165		21.0 (14.2, 27.8)	138		29.0 (24.4, 33.6)	372		43.5 (36.8, 50.1)	214	
Family annual income (yuan)			**<0.001**			**<0.001**			**0.049**			0.281
0–50,000	2.5 (2.0, 3.1)	3,076		10.6 (9.3, 11.8)	2,377		25.2 (23.6, 26.7)	3,082		42.3 (40.0, 44.5)	1888	
50,000–100,000	4.6 (3.0, 6.2)	650		17.1 (14.4, 19.8)	754		24.7 (21.8, 27.5)	860		38.4 (34.2, 42.6)	516	
>100,000	9.0 (6.1, 11.9)	377		20.5 (17.3, 23.8)	594		29.8 (26.1, 33.5)	588		41.7 (36.1, 47.2)	300	

Abbreviations: 95%CIs, 95% confident intervals.

Note:.

^a^ Preterm birth referred to a child born at a gestational age greater than 28 weeks and less than 36 weeks.

After stratification, a higher prevalence of VDD was more likely among girls (Supplementary Figure S3), Han children/adolescents, those breastfed for over 6 months (OR = 1.429), urban residents (OR = 0.587), obese individuals (OR = 1.796), and those with an annual income over 100,000 yuan (OR = 1.438) (P < 0.05). These findings are detailed in Supplementary Table S6.

### The Prevalence of VDD in Different Season

The prevalence of VDD varied across seasons by age group. For children aged 0–3 years, the highest VDD prevalence was in autumn (14.1%). Among 4–6 years, VDD was more common in autumn (22.9%) and winter (11.5%) than in summer (5.9%) and spring (5.8%). For ages 7–12 years and 13–18 years, most 25(OH)D measurements were taken in autumn and winter, with limited data for spring and summer (Supplementary Table S7).

### Climate-Specific Zone-Stratified Prevalence of VDD

The highest prevalence of VDD among preschoolers aged 4–6 years was found in semiarid and subhumid zones, at 12.8%. For middle childhood (7–12 years), the VDD prevalence was 34.4%, and for teenagers (13–18 years), it was 41.7%. Toddlers (0–3 years) had the highest VDD prevalence in subhumid zones at 2.6%. The lowest prevalence was observed in semiarid and subhumid zones for toddlers (0.8%) and in the subhumid zone for middle childhood (5.3%) and teenagers (37.1%). Due to limited samples, the lowest VDD prevalence for preschoolers was 4.2% in three zones (humid, mountainous humid, and semiarid) (P < 0.05) (Table 5).

**TABLE 5:** Climate-specific zone stratified prevalence of 25-hydroxyvitamin D deficiency across age groups (China, 2022).

Climate-specific zones	0–3 years			4–6 years			7–12 years			13–18 years		
	Prevalence (%) (95% CI)	Testing sample	*P*	Prevalence (%) (95% CI)	Testing sample	*P*	Prevalence (%) (95% CI)	Testing sample	*P*	Prevalence (%) (95% CI)	Testing sample	*P*
			<0.001			<0.001			<0.001			0.033
Humid zone	2.4 (1.4, 3.4)	926		4.2 (2.7, 5.7)	691		28.4 (24.3, 32.5)	458		38.9 (34.2, 43.6)	414	
Sub-humid zone	2.6 (0.6, 4.7)	227		9.6 (5.9, 13.3)	250		5.3 (0.9, 24.6)	19		37.1 (29.1, 45.0)	143	
Mountainous humid zone	2.4 (1.4, 3.4)	926		4.2 (2.7, 5.7)	691		28.4 (24.3, 32.5)	458		38.9 (34.2, 43.6)	414	
Semi-arid and sub-humid zone	0.8 (0.3, 2.5)	353		12.8 (8.0, 17.6)	187		34.4 (30.5, 38.4)	549		41.7 (36.7, 46.7)	379	
Semi-arid zone	2.4 (1.4, 3.4)	926		4.2 (2.7, 5.7)	691		28.4 (24.3, 32.5)	458		38.9 (34.2, 43.6)	414	

## Discussion

This cross-sectional study found that lower serum levels of total vitamin D, as well as vitamins D2 and D3, were more common among older adolescents compared to young children, corresponding with a higher prevalence of VDD in older adolescents. High-risk groups for low vitamin D and elevated VDD included girls, Han individuals, urban residents, those with obesity, those breastfed for over 6 months, and families with annual incomes over 100,000 yuan. Lower vitamin D levels were mainly seen in humid and subhumid zones, while VDD prevalence was highest in semiarid and subhumid regions.

Numerous studies have shown that VDD is common among children and adolescents, particularly in older teens [25]. A multi-center study in Hainan Province found the highest serum 25(OH)D levels in the 0–1 year group and the lowest in the 14–18 years group [26]. Similarly, a cross-sectional study of 1,510 adolescents (12–18 years) from the Korea National Health and Nutrition Examination Survey (KNHANES) 2008–2009 reported an age-related decline in 25(OH)D [27]. Notably, similar results were also observed in South Africa, which possesses a tropical climate. Newborns exhibit the lowest prevalence of VDD, whereas youth aged 11–20 years show the highest prevalence [28].

This age-related decline in vitamin D status may be partly attributed to the increased utilization of vitamin D by soft tissue during growth [29], and a decreased intake of vitamin D among children with their age due to an unhealthy diet lacking dairy products and insufficient parental oversight [30, 31]. Moreover, the capacity for cutaneous to synthesize 25(OH)D3 decreases with age [32]. Another plausible explanation for the poorer vitamin D status observed in older children is the reduced time available for outdoor play as their educational commitments increase, leading to more time spent indoors [8]. This age-related decline in physical activity and sunlight exposure consequently impacts the levels of 25(OH)D3, the primary source of serum vitamin D synthesized naturally in human skin upon exposure to UVB rays from sunlight [4, 33]. Therefore, parents need to pay more attention to children’s vitamin D supplementation and increase outdoor activities to increase vitamin production. The Chinese Medical Association recommends a daily vitamin D supply of 400 IU for children aged 0–1 year, and 400–800 IU for children over 1 year of age [34]. In addition, scientific research has shown that short-term exposure to sunlight (e.g., 2-3 times a week, 15–30 min each time) is sufficient to achieve maximum vitamin D synthesis. Beyond this time, although vitamin D synthesis may continue, the increase is relatively limited and the risk increases accordingly [35].

Consistent with findings from other studies [25, 27, 36–39], we found that lower vitamin D levels and higher VDD prevalence were more pronounced in girls than boys. This gender disparity may be linked to lifestyle differences. Due to a preference for lighter skin, girls are more likely to use high sun protection factor (SPF) sunscreen, wear protective clothing, and limit outdoor activities during sun exposure to prevent tanning or sunburn. These practices may significantly reduce the cutaneous synthesis of 25(OH)D3. Sufficient UV radiation exposure necessary for generating sufficient serum 25(OH)D levels typically requires sun exposure of one-fourth of the body’s skin for 10–15 min without sunscreen protection [40].

Additionally, low vitamin D status appeared to be more prevalent among Han Chinese compared to other ethnic groups. This disparity may be partly due to genetic differences affecting vitamin D3 synthesis. Furthermore, compared to their urban counterparts, ethnic minorities in China predominantly reside in rural, underdeveloped, and mountainous regions [41]. These minorities may engage in outdoor activities more frequently due to their unique geographical locations, customs, and cultural practices. This could help explain the variations in vitamin D status observed across different ethnicities and living environments in Hainan.

Our study found a strong link between obesity and a high prevalence of VDD, aligning with meta-analysis findings that indicate a 35% increased risk of VDD in obese individuals compared to those with normal weight [42]. The reasons for lower vitamin D levels in obesity are unclear, but a key hypothesis is that fat-soluble vitamin D may be sequestered in adipose tissue, impairing absorption [43]. Excess body fat can also disrupt hormonal pathways essential for skeletal health. For instance, leptin, a hormone from fat cells, may inhibit the renal synthesis of active vitamin D [44]. A biologically plausible explanation for the correlation between low circulating vitamin D levels and elevated body fat is that vitamin D plays a role in regulating adipogenesis [45]. Vitamin D is a group of fat-soluble secosteroids, and its bioavailability is diminished due to sequestration in body fat and volumetric dilution [46, 47]. Furthermore, the social stigma associated with obesity may lead obese children to limit their exposure to sunlight and outdoor activities A more sedentary lifestyle, coupled with the tendency to wear darker clothing to cover their bodies, greatly restricts the synthesis of vitamin D in the skin [42].

Although breast milk is considered the best nutrition for infants, excessive breastfeeding without vitamin D supplementation poses a risk for VDD and rickets in infants [48]. This may explain why children exclusively breastfed for more than 6 months exhibited lower serum vitamin D levels and a higher prevalence of VDD in our study. The median concentration of vitamin D in human milk and colostrum is only 15.9 ± 8.6 IU/L [49]. However, according to the expert consensus on clinical application of vitamin D in Chinese children, vitamin D supplementation should be started within the first week after birth until the age of 18 to ensure the growth and fertility needs of infants, young children, and adolescents [34]. Thus, breastfeeding alone is insufficient to provide adequate circulating levels of precursor hormone 25(OH)D for infants [50]. Due to the limited transfer rate, breast milk contains only 5–136 IU/L vitamin D, despite maternal intake of 600–700 IU/D [51]. A randomized controlled trial (RCT) involving 334 mother-infants pairs further demonstrated that maternal vitamin D supplementation of 6400 IU/day could facilitate sufficient vitamin D transfer in breast milk to meet the needs of her nursing infants [52]. Our findings suggest that appropriate vitamin D supplementation for breastfeeding infants is crucial and should be advised for lactating mothers.

While it may seem intuitive that higher-income families would have better vitamin D levels due to greater access to nutrient-rich foods, evidence is mixed regarding the actual vitamin D status across different income groups. Our study indicates that low circulating vitamin D levels and high prevalence of VDD are more common in children and adolescents from affluent families compared to those from lower-income households, though the impact of income on vitamin D status remains inconclusive. For example, *Weng FL et al.* [53] reported that low vitamin D status was more prevalent among family members with lower annual incomes. But studies have shown that high-income families may be more inclined to consume fast food and ready to eat foods, which often lack necessary nutrients, including vitamin D [54]. In addition, high-income families may reduce their time spent outdoors due to work and life pressures. Especially in urban environments, many high-income families may live in high-rise buildings with relatively fewer opportunities for outdoor activities. This lifestyle change may make them more susceptible to vitamin D deficiency issues [55]. Further research is needed to assess whether family income is an independent predictor of vitamin D status.

Vitamin D production can vary significantly by latitude, despite differences in participant age. A comprehensive study in China found that only Hainan Province had a serum 25(OH)D concentration exceeding 30 ng/mL, with a low prevalence of VDD at 7.3% [56]. Hainan, located near the equator, receive more sunlight than regions closer to the poles; however, the amount of UVB radiation varies by region [32]. Ozone in the atmosphere absorbs more UVB radiation as the path length increases due to the sun’s oblique angle at zenith, further diminishing the amount of UVB rays that reach the Earth’s surface [4]. Consequently, the energy of UVB radiation may be insufficient to promote the conversion of 7-dehydrocholesterol to previtamin D3 on rainy or foggy days, thereby limiting the transformation of previtamin D3 into vitamin D3 in the skin [57]. Particularly, sunlight exposure can also be limited in regions characterized by high humidity and a sultry climate, where sun-seeking behavior is uncommon, even during the summertime in Hainan. Local residents often avoid the heat and sun by remaining indoors or adopting specific clothing practices. phenomenon may help explain the vitamin D status in some humid, subhumid, and humid zones. This phenomenon is not unique to China; it is also observed in countries blessed with abundant sunlight, like Malaysia and numerous nations across the African continent, and South Asia [58, 59], VDD remains common, suggesting that sunlight alone is insufficient to ensure adequate vitamin D levels. Factors such as social culture, lifestyle, and dietary habits also play a significant role in determining vitamin D status [60].

Our study has several important limitations. First, the generalizability of our findings may be limited, as our sample consisted solely of Chinese children and adolescents. However, this provincial-level sample was substantial and representative, encompassing healthy children and adolescents across a broad age range (0–18 years), suggesting that the results could be relevant in similar settings. Second, blood samples were collected during the autumn and winter months, which may have influenced 25(OH)D levels, potentially resulting in higher vitamin D concentrations than would be expected during other times of the year. Third, due to the cross-sectional design of this study, we were unable to fully control for all potential confounding factors. While we adjusted for several demographic and health-related covariates, such as age, sex, nationality, BMI, prenatal vitamin D supplementation, and exclusive breastfeeding duration, we did not adequately account for other possible confounders like dietary intake and individual sunlight exposure habits. These factors could significantly influence serum vitamin D levels, and their lack of direct adjustment in our analysis may have introduced bias. Finally, as this is an observational study, causal inferences cannot be made, and our findings should be interpreted with caution.

### Conclusion

In summary, we demonstrated that low vitamin D status and VDD were more prevalent among older children and adolescents. Vitamin D status was particularly poor in girls, Han Chinese individuals, those diagnosed with obesity, those who were excessively breastfed, and those from affluent families. The humid geographic location may be associated with VDD. The study addresses a significant gap in the understanding of vitamin D status and VDD prevalence among children and teenagers residing in southernmost China. Consequently, the underlying causes of VDD among different demographics can be further investigated. Comprehensive breastfeeding guidelines should integrate vitamin D supplementation recommendations, highlight the need for targeted interventions for high-risk groups, promote educational campaigns on vitamin D’s importance, clarify sun exposure safety, and encourage dietary improvements. Such an encompassing strategy will not only help prevent vitamin D deficiency in infants and children but will also foster long-term health benefits.
