# Supplementary material for: Vitamin D Deficiency and Associated Factors in Children: A Multicenter Study of 21,811 Samples in Southern China
Source: Int J Public Health. 2025 Jan 6;69:1607411. doi: 10.3389/ijph.2024.1607411 (PMC11742945; doi:10.3389/ijph.2024.1607411)
Supplement: Supplementary file 1 [file DataSheet1.docx]

Supplementary Material

# Supplementary Figures


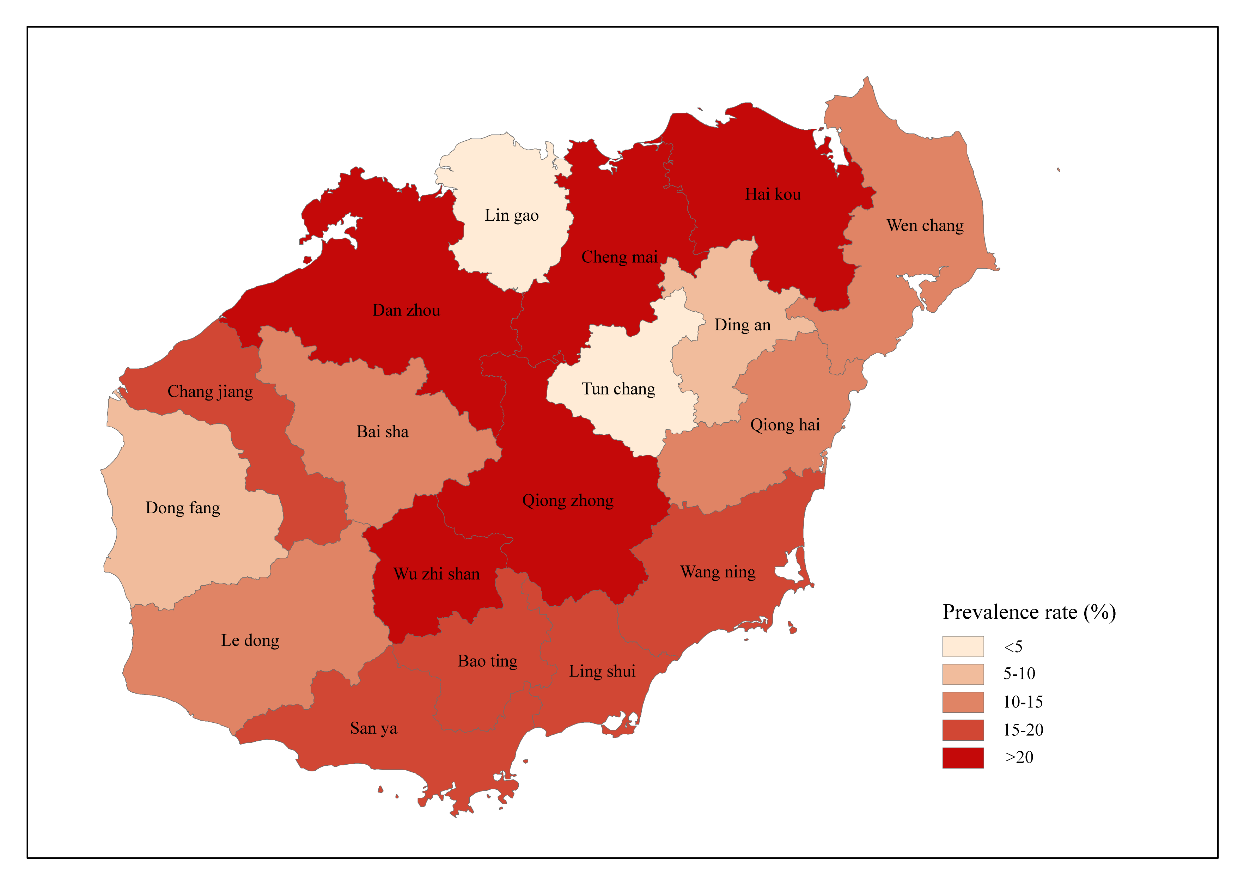


**Supplementary Figure 1.** The prevalence of 25-hydroxyvitamin D among Hainan cities **(China, 2022)**.





**Supplementary Figure 2.** The prevalence of 25-hydroxyvitamin D among Hainan cities by age groups **(China, 2022)**.

**
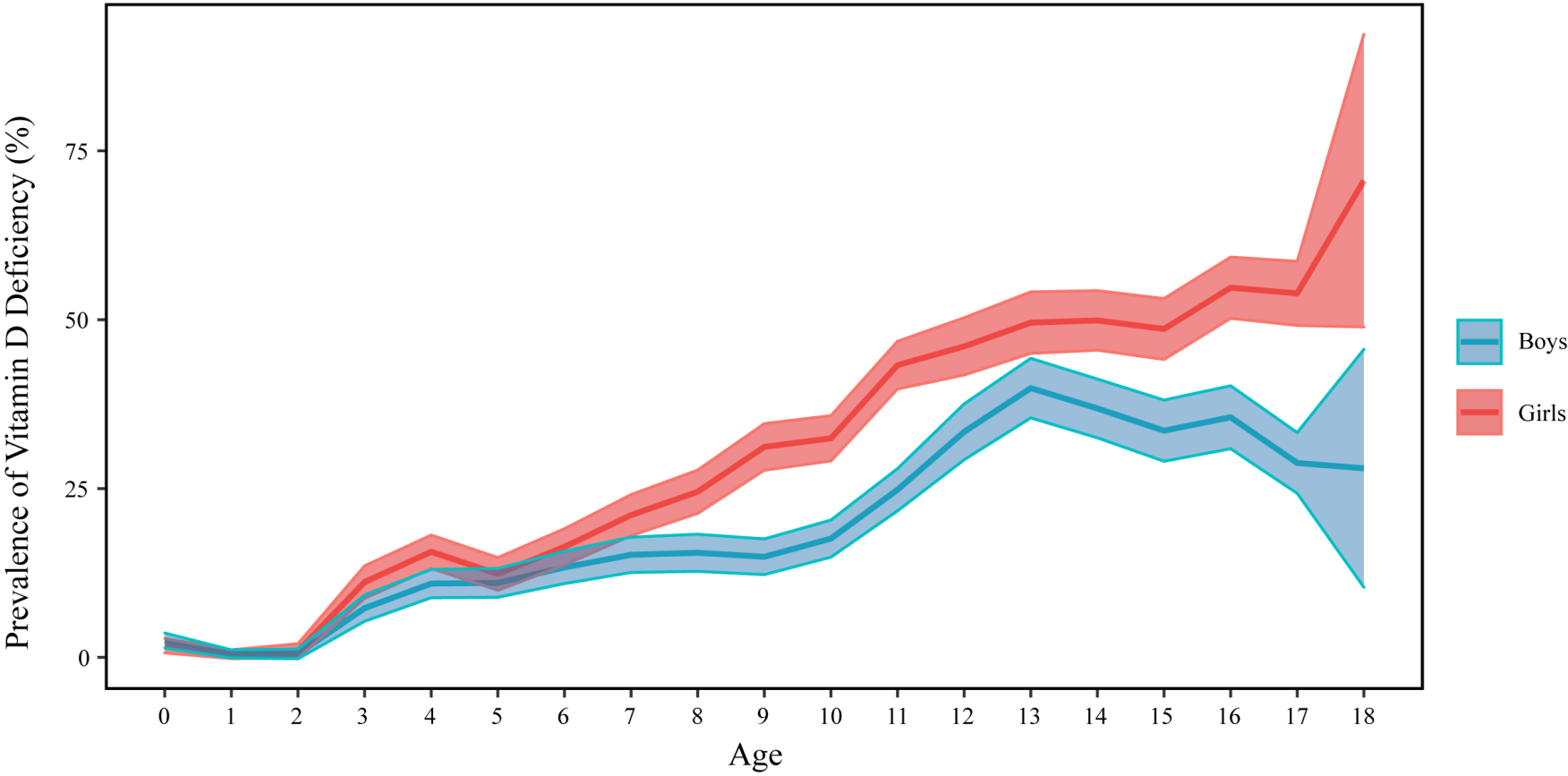
**

**Supplementary Figure 3.** The sex-specified prevalence of 25-hydroxyvitamin D deficiency across age groups **(China, 2022)**.

# Supplementary Tables

**Supplementary Table 1.** The detection rate of 25-hydroxyvitamin D across age groups **(China, 2022)**.

| **Covariates** | **0-3 y** | |  | **4-6 y** | |  | **7-12 y** | |  | **13-18 y** | |
| --- | --- | --- | --- | --- | --- | --- | --- | --- | --- | --- | --- |
|  | **Study**  **sample (%)** | **Total sample (%)** |  | **Study**  **sample (%)** | **Total sample (%)** |  | **Study**  **sample (%)** | **Total sample (%)** |  | **Study**  **sample (%)** | **Total sample (%)** |
| **Overall** | 4382 | 8612 |  | 4734 | 11430 |  | 8177 | 43234 |  | 4518 | 13555 |
| **Gender** |  |  |  |  |  |  |  |  |  |  |  |
| Male | 54.8 (53.4, 56.3) | 54.9 (53.8, 55.9) |  | 51.9 (50.5, 53.3) | 52.1 (51.1, 53.0) |  | 49.6 (48.6, 50.7) | 51.0 (50.5, 51.5) |  | 48.3 (46.8, 49.8) | 49.4 (48.5, 50.2) |
| Female | 45.2 (43.7, 46.6) | 45.1 (44.1, 46.2) |  | 48.1 (46.7, 49.5) | 47.9 (47.0, 48.9) |  | 50.4 (49.3, 51.4) | 49.0 (48.5, 49.5) |  | 51.7 (50.2, 53.2) | 50.6 (49.8, 51.5) |
| **Nationalities** |  |  |  |  |  |  |  |  |  |  |  |
| Han | 76.4 (75.1, 77.7) | 74.0 (73.1, 75.0) |  | 82.6 (81.4, 83.7) | 83.8 (83.0, 84.6) |  | 83.3 (82.2, 84.4) | 85.0 (84.6, 85.5) |  | 80.3 (78.8, 81.8) | 84.2 (83.3, 85.0) |
| Li | 21.0 (19.7, 22.2) | 23.0 (22.1, 23.9) |  | 15.1 (14.0, 16.2) | 14.2 (13.5, 15.0) |  | 15.2 (14.1, 16.2) | 12.9 (12.5, 13.4) |  | 16.1 (14.7, 17.5) | 13.5 (12.7, 14.2) |
| Others | 2.6 (2.2, 3.1) | 2.9 (2.6, 3.3) |  | 2.3 (1.9, 2.8) | 2.0 (1.7, 2.3) |  | 1.5 (1.2, 1.9) | 2.0 (1.8, 2.2) |  | 3.6 (2.9, 4.3) | 2.4 (2.0, 2.7) |
| **Regions** |  |  |  |  |  |  |  |  |  |  |  |
| Urban | 34.6 (33.1, 36.0) | 39.0 (37.9, 40.0) |  | 55.4 (54.0, 56.8) | 47.5 (46.6, 48.4) |  | 74.6 (73.7, 75.6) | 38.3 (37.9, 38.8) |  | 77.8 (76.6, 79.1) | 56.5 (55.6, 57.3) |
| Rural | 64.4 (62.9, 65.8) | 60.4 (59.3, 61.4) |  | 43.4 (42.0, 44.8) | 51.7 (50.8, 52.6) |  | 25.4 (24.4, 26.3) | 61.6 (61.2, 62.1) |  | 22.1 (20.9, 23.3) | 43.3 (42.4, 44.1) |
| **Quartile of BMI (kg/m2)** |  |  |  |  |  |  |  |  |  |  |  |
| Q1 | 25.1 (24.6, 26.2) | 24.0 (23.5, 25.1) |  | 50.2 (49.6, 51.4) | 46.5 (45.9, 47.7) |  | 27.5 (27.0, 28.6) | 27.4 (26.9, 28.5) |  | 1.5 (1.3, 1.7) | 1.2 (1.1, 1.5) |
| Q2 | 40.7 (40.1, 41.9) | 34.9 (34.3, 36.1) |  | 32.6 (32.0, 33.7) | 33.4 (32.8, 34.5) |  | 25.5 (25.0, 26.6) | 27.4 (26.9, 28.5) |  | 6.9 (6.6, 7.5) | 5.1 (4.8, 5.6) |
| Q3 | 30.2 (29.6, 31.3) | 34.0 (33.4, 35.1) |  | 12.9 (12.4, 13.7) | 15.3 (14.8, 16.1) |  | 23.7 (23.1, 24.7) | 24.5 (24.0, 25.6) |  | 31.4 (30.8, 32.5) | 28.3 (27.7, 29.3) |
| Q4 | 3.9 (3.7, 4.4) | 7.1 (6.8, 7.7) |  | 4.3 (4.1, 4.8) | 4.8 (4.6, 5.4) |  | 23.2 (22.7, 24.2) | 20.6 (20.1, 21.6) |  | 60.2 (59.5, 61.4) | 65.4 (64.8, 66.6) |
| **Vitamin D supplement during gestation** |  |  |  |  |  |  |  |  |  |  |  |
| Never | 75.3 (74.7, 76.3) | 74.2 (73.6, 75.3) |  | 64.9 (64.3, 66.1) | 49.1 (48.5, 50.3) |  | 46.2 (45.6, 47.4) | 43.8 (43.2, 45.0) |  | 41.8 (41.2, 43.0) | 40.7 (40.0, 41.8) |
| Seldom | 8.7 (8.3, 9.4) | 10.1 (9.7, 10.8) |  | 25.7 (25.1, 26.7) | 41.6 (41.0, 42.8) |  | 44.8 (44.2, 46.0) | 47.2 (46.5, 48.4) |  | 46.7 (46.0, 47.8) | 48.5 (47.8, 49.7) |
| Often | 4.7 (4.4, 5.2) | 4.8 (4.5, 5.3) |  | 4.8 (4.5, 5.3) | 6.5 (6.1, 7.1) |  | 6.9 (6.6, 7.5) | 7.1 (6.8, 7.7) |  | 9.3 (8.9, 10.0) | 9.0 (8.6, 9.7) |
| Always | 11.3 (10.9, 12.1) | 10.9 (10.5, 11.6) |  | 4.6 (4.3, 5.1) | 2.8 (2.6, 3.2) |  | 2.1 (1.9, 2.4) | 1.9 (1.7, 2.2) |  | 2.2 (2.0, 2.6) | 1.9 (1.7, 2.2) |
| **Premature birth ^a^** |  |  |  |  |  |  |  |  |  |  |  |
| Yes | 7.8 (7.0, 8.7) | 7.6 (7.0, 8.1) |  | 8.8 (7.8, 9.7) | 9.9 (9.2, 10.6) |  | 9.7 (8.8, 10.7) | 14.7 (14.2, 15.2) |  | 15.7 (14.2, 17.2) | 20.5 (19.5, 21.5) |
| No | 92.2 (91.3, 93.0) | 92.4 (91.9, 93.0) |  | 91.2 (90.3, 92.2) | 90.1 (89.4, 90.8) |  | 90.3 (89.3, 91.2) | 85.3 (84.8, 85.8) |  | 84.3 (82.8, 85.8) | 79.5 (78.5, 80.5) |
| **Conception ways** |  |  |  |  |  |  |  |  |  |  |  |
| Natural pregnancy | 97.9 (97.5, 98.3) | 98.0 (97.7, 98.3) |  | 97.9 (97.4, 98.3) | 98.0 (97.7, 98.3) |  | 98.4 (98.1, 98.8) | 98.2 (98.0, 98.4) |  | 98.9 (98.5, 99.3) | 98.9 (98.7, 99.2) |
| Assisted reproduction | 2.1 (1.7, 2.5) | 2.0 (1.7, 2.3) |  | 2.1 (1.7, 2.6) | 2.0 (1.7, 2.3) |  | 1.6 (1.2, 1.9) | 1.8 (1.6, 2.0) |  | 1.1 (0.7, 1.5) | 1.1 (0.8, 1.3) |
| **Delivery methods** |  |  |  |  |  |  |  |  |  |  |  |
| Natural Childbirth | 71.0 (69.6, 72.4) | 70.8 (69.8, 71.8) |  | 71.3 (69.9, 72.7) | 71.7 (70.7, 72.7) |  | 73.3 (72.0, 74.6) | 77.1 (76.5, 77.6) |  | 82.1 (80.7, 83.6) | 84.7 (83.8, 85.5) |
| Cesarean section | 29.0 (27.6, 30.4) | 29.2 (28.2, 30.2) |  | 28.7 (27.3, 30.1) | 28.3 (27.3, 29.3) |  | 26.7 (25.4, 28.0) | 22.9 (22.4, 23.5) |  | 17.9 (16.4, 19.3) | 15.3 (14.5, 16.2) |
| **Number of births** |  |  |  |  |  |  |  |  |  |  |  |
| Single birth | 95.6 (95.0, 96.2) | 96.3 (95.9, 96.7) |  | 93.6 (92.8, 94.3) | 94.0 (93.4, 94.5) |  | 94.6 (93.9, 95.2) | 93.7 (93.4, 94.0) |  | 94.8 (93.9, 95.6) | 94.0 (93.5, 94.6) |
| Multiple births | 4.4 (3.8, 5.0) | 3.7 (3.3, 4.1) |  | 6.4 (5.7, 7.2) | 6.0 (5.5, 6.6) |  | 5.4 (4.8, 6.1) | 6.3 (6.0, 6.6) |  | 5.2 (4.4, 6.1) | 6.0 (5.4, 6.5) |
| **Birth weight (kg)** |  |  |  |  |  |  |  |  |  |  |  |
| <2.5 | 7.1 (6.3, 7.8) | 6.6 (6.0, 7.1) |  | 5.5 (4.8, 6.2) | 5.0 (4.5, 5.5) |  | 5.3 (4.6, 6.0) | 5.2 (4.9, 5.5) |  | 6.4 (5.4, 7.4) | 6.4 (5.8, 7.0) |
| 2.5-4.0 | 87.3 (86.3, 88.3) | 87.9 (87.2, 88.6) |  | 82.7 (81.5, 83.9) | 80.7 (79.8, 81.6) |  | 80.4 (79.2, 81.6) | 75.9 (75.3, 76.5) |  | 73.9 (72.2, 75.7) | 72.3 (71.3, 73.4) |
| >4.0 | 5.6 (4.9, 6.3) | 5.6 (5.1, 6.1) |  | 11.8 (10.8, 12.8) | 14.2 (13.4, 15.0) |  | 14.3 (13.2, 15.3) | 18.9 (18.4, 19.5) |  | 19.7 (18.1, 21.3) | 21.3 (20.3, 22.3) |
| **Exclusively breastfed for 6 months or more** |  |  |  |  |  |  |  |  |  |  |  |
| Yes | 56.7 (55.2, 58.2) | 55.5 (54.4, 56.5) |  | 79.8 (78.5, 81.0) | 83.2 (82.4, 84.1) |  | 86.1 (85.1, 87.1) | 88.1 (87.7, 88.6) |  | 92.6 (91.6, 93.6) | 93.2 (92.7, 93.8) |
| No | 43.3 (41.8, 44.8) | 44.5 (43.5, 45.6) |  | 20.2 (19.0, 21.5) | 16.8 (15.9, 17.6) |  | 13.9 (12.9, 14.9) | 11.9 (11.4, 12.3) |  | 7.4 (6.4, 8.4) | 6.8 (6.2, 7.3) |
| **Growth and development assessment** |  |  |  |  |  |  |  |  |  |  |  |
| Retardation | 15.6 (14.4, 16.7) | 17.3 (16.4, 18.1) |  | 14.7 (13.6, 15.7) | 13.5 (12.8, 14.2) |  | 7.5 (6.9, 8.1) | 10.6 (10.3, 10.9) |  | 7.4 (6.7, 8.2) | 8.8 (8.2, 9.3) |
| Normal | 80.1 (78.8, 81.4) | 76.1 (75.1, 77.0) |  | 82.0 (80.8, 83.2) | 82.9 (82.1, 83.6) |  | 87.6 (86.9, 88.4) | 85.6 (85.3, 86.0) |  | 87.6 (86.6, 88.6) | 86.9 (86.3, 87.5) |
| Obesity | 4.3 (3.7, 5.0) | 6.7 (6.1, 7.3) |  | 3.3 (2.8, 3.9) | 3.6 (3.2, 4.0) |  | 4.8 (4.4, 5.3) | 3.8 (3.6, 4.0) |  | 4.9 (4.3, 5.6) | 4.3 (4.0, 4.7) |
| **Family annual income (yuan)** |  |  |  |  |  |  |  |  |  |  |  |
| 0-50,000 | 75.0 (73.6, 76.3) | 74.9 (74.0, 75.9) |  | 63.8 (62.3, 65.4) | 67.5 (66.4, 68.5) |  | 68.0 (66.7, 69.4) | 77.7 (77.1, 78.2) |  | 69.8 (68.1, 71.6) | 76.8 (75.8, 77.7) |
| 50,000-100,000 | 15.8 (14.7, 17.0) | 16.1 (15.3, 16.9) |  | 20.2 (19.0, 21.5) | 19.4 (18.5, 20.3) |  | 19.0 (17.8, 20.1) | 15.1 (14.6, 15.6) |  | 19.1 (17.6, 20.6) | 15.0 (14.2, 15.8) |
| ＞100,000 | 9.2 (8.3, 10.1) | 8.9 (8.3, 9.5) |  | 15.9 (14.8, 17.1) | 13.1 (12.4, 13.9) |  | 13.0 (12.0, 14.0) | 7.2 (6.9, 7.6) |  | 11.1 (9.9, 12.3) | 8.2 (7.6, 8.9) |

**Supplementary Table 2.** The region-specified levels of 25-hydroxyvitamin D across age groups **(China, 2022).**

| **Regions** | **0-3 y** | | |  | **4-6 y** | | |  | **7-12 y** | | |  | **13-18 y** | | |
| --- | --- | --- | --- | --- | --- | --- | --- | --- | --- | --- | --- | --- | --- | --- | --- |
|  | **25(OH)D2** | **25(OH)D3** | **25(OH)D ^a^** |  | **25(OH)D2** | **25(OH)D3** | **25(OH)D ^a^** |  | **25(OH)D2** | **25(OH)D3** | **25(OH)D ^a^** |  | **25(OH)D2** | **25(OH)D3** | **25(OH)D ^a^** |
| Haikou | 0.51 (0.31, 0.70) | 28.20 (23.73, 33.52) | 28.98 (24.28, 34.10) |  | 0.44 (0.23, 0.68) | 24.13 (19.90, 28.40) | 24.67 (20.40, 28.89) |  | 0.40 (0.19, 0.59) | 22.67 (18.99, 26.70) | 23.08 (19.42, 27.12) |  | 0.40 (0.20, 0.61) | 20.47 (16.53, 23.84) | 20.97 (16.88, 24.32) |
| Ding'an County | 0.30 (0.14, 0.60) | 39.64 (31.90, 47.66) | 39.74 (31.97, 47.73) |  | 0.24 (0.13, 0.42) | 26.75 (24.44, 31.11) | 27.05 (24.52, 31.55) |  | 0.31 (0.17, 0.40) | 25.29 (19.72, 28.78) | 25.60 (20.04, 28.86) |  | 0.36 (0.31, 0.44) | 21.02 (17.69, 24.62) | 21.34 (18.03, 25.14) |
| Danzhou | 0.60 (0.41, 0.72) | 32.93 (26.83, 40.27) | 33.64 (27.44, 41.44) |  | 0.59 (0.43, 0.83) | 24.81 (21.49, 28.45) | 25.52 (22.05, 29.20) |  | 0.35 (0.16, 0.57) | 20.22 (18.31, 24.24) | 20.52 (18.74, 24.64) |  | 0.33 (0.20, 0.53) | 18.55 (15.68, 21.63) | 18.97 (16.00, 21.93) |
| Lingao County | 0.28 (0.18, 0.54) | 41.89 (34.65, 51.58) | 41.89 (34.88, 51.68) |  | 0.35 (0.20, 0.56) | 27.96 (23.98, 31.98) | 28.48 (24.17, 32.26) |  | - | - | - |  | - | - | - |
| Chengmai County | 0.56 (0.33, 0.74) | 35.30 (27.89, 47.33) | 35.82 (28.34, 47.33) |  | 0.47 (0.21, 0.58) | 28.35 (24.11, 33.85) | 28.75 (24.24, 34.32) |  | 0.44 (0.37, 0.50) | 22.42 (18.56, 26.64) | 22.76 (19.11, 26.84) |  | 0.31 (0.16, 0.47) | 17.62 (15.36, 21.26) | 18.06 (15.59, 21.67) |
| Tunchang County | 0.38 (0.07, 0.74) | 41.79 (35.21, 46.79) | 41.79 (35.36, 46.79) |  | 0.56 (0.25, 0.78) | 28.26 (24.30, 33.60) | 28.55 (24.75, 34.82) |  | 0.50 (0.39, 0.69) | 26.10 (22.87, 29.61) | 26.40 (23.34, 30.12) |  | - | - | - |
| Wenchang | 0.49 (0.34, 0.83) | 38.08 (32.88, 43.12) | 38.15 (32.98, 43.20) |  | 0.49 (0.23, 0.67) | 28.87 (24.16, 34.11) | 29.66 (24.65, 34.87) |  | 0.23 (0.12, 0.51) | 20.49 (17.29, 24.44) | 20.77 (17.48, 24.88) |  | 0.50 (0.21, 0.65) | 21.26 (18.60, 24.41) | 21.62 (18.78, 24.80) |
| Qionghai | 0.34 (0.23, 0.48) | 35.97 (30.07, 42.45) | 36.24 (30.32, 42.71) |  | 0.32 (0.18, 0.48) | 27.32 (23.96, 31.94) | 27.86 (24.30, 32.36) |  | 0.34 (0.24, 0.47) | 20.54 (18.39, 24.64) | 20.57 (18.88, 25.08) |  | 0.47 (0.28, 0.58) | 20.82 (17.94, 24.87) | 21.30 (18.51, 25.13) |
| Wanning | 0.44 (0.21, 0.63) | 31.85 (27.72, 36.96) | 32.21 (28.27, 37.17) |  | 0.44 (0.23, 0.62) | 29.93 (26.20, 33.55) | 30.39 (26.50, 34.01) |  | 0.39 (0.27, 0.55) | 24.00 (20.07, 28.08) | 24.40 (20.44, 28.66) |  | 0.35 (0.22, 0.47) | 20.57 (17.77, 23.57) | 21.04 (18.06, 23.92) |
| Changjiang Li-AC | 0.65 (0.33, 0.89) | 35.06 (30.09, 40.28) | 35.15 (30.47, 40.44) |  | 0.52 (0.14, 0.73) | 27.04 (23.94, 31.53) | 27.62 (24.53, 31.62) |  | 0.49 (0.24, 0.68) | 29.46 (22.29, 31.11) | 29.97 (22.78, 31.32) |  | 0.46 (0.37, 0.62) | 20.72 (17.68, 24.15) | 21.23 (18.18, 24.54) |
| Dongfang | 0.36 (0.19, 0.57) | 29.40 (25.27, 37.00) | 29.94 (25.59, 37.79) |  | 0.40 (0.19, 0.64) | 26.50 (22.89, 30.64) | 27.07 (23.20, 31.22) |  | - | - | - |  | - | - | - |
| Sanya | 0.51 (0.36, 0.70) | 35.66 (29.66, 45.00) | 35.73 (29.96, 45.00) |  | 0.53 (0.41, 0.88) | 25.44 (22.33, 29.17) | 26.24 (22.94, 29.92) |  | 0.50 (0.32, 0.74) | 22.93 (19.58, 26.26) | 23.42 (20.08, 26.86) |  | 0.47 (0.26, 0.64) | 20.68 (17.25, 24.82) | 21.20 (17.50, 25.44) |
| Lingshui Li -AC | 0.30 (0.19, 0.55) | 34.49 (29.05, 44.06) | 34.91 (29.24, 44.06) |  | 0.30 (0.16, 0.50) | 28.35 (24.34, 33.07) | 28.70 (24.78, 33.50) |  | 0.46 (0.38, 0.62) | 18.93 (16.38, 26.42) | 19.50 (17.16, 26.70) |  | 0.40 (0.29, 0.54) | 19.50 (16.22, 22.87) | 19.84 (16.70, 23.44) |
| Ledong Li-AC | 0.58 (0.24, 0.95) | 35.89 (30.93, 41.35) | 35.95 (31.04, 41.35) |  | 0.56 (0.38, 0.76) | 26.04 (22.94, 30.20) | 26.62 (23.45, 30.55) |  | 0.64 (0.51, 0.75) | 25.73 (22.20, 29.77) | 26.26 (22.60, 30.45) |  | 0.31 (0.15, 0.53) | 24.05 (17.83, 28.72) | 24.10 (18.28, 28.76) |
| Baoting Li and Miao-AC | 0.28 (0.17, 0.43) | 35.74 (26.69, 51.95) | 35.92 (27.03, 51.95) |  | 0.35 (0.21, 0.55) | 26.86 (23.63, 30.91) | 27.28 (23.95, 31.37) |  | 0.44 (0.32, 0.58) | 24.82 (21.05, 29.11) | 25.24 (21.40, 29.54) |  | 0.26 (0.11, 0.49) | 20.51 (17.51, 23.25) | 20.71 (17.78, 23.94) |
| Wuzhishan | 0.61 (0.38, 1.14) | 35.64 (28.79, 41.72) | 37.50 (29.72, 45.74) |  | 0.87 (0.50, 1.06) | 27.01 (23.83, 29.80) | 27.98 (24.88, 30.68) |  | 0.33 (0.18, 0.73) | 23.40 (19.51, 26.52) | 23.76 (19.96, 27.20) |  | 0.22 (0.13, 0.32) | 17.72 (15.84, 20.81) | 18.12 (16.01, 20.92) |
| Qiongzhong Li and Miao-AC | 0.54 (0.38, 0.81) | 37.87 (28.47, 50.57) | 37.95 (28.98, 50.57) |  | 0.38 (0.29, 0.59) | 24.62 (20.55, 28.17) | 25.12 (21.16, 28.80) |  | 0.44 (0.35, 0.59) | 20.28 (16.81, 24.64) | 20.93 (17.20, 25.06) |  | 0.40 (0.29, 0.69) | 22.23 (18.36, 26.08) | 22.88 (18.76, 26.58) |
| Baisha Li-AC | - | - | - |  | - | - | - |  | - | - | - |  | 0.46 (0.27, 0.70) | 24.69 (19.06, 27.78) | 25.08 (19.58, 28.56) |

**Abbreviations:** AC, autonomous county.

**Note:** Levels of 25(OH)D2(ng/ml), 25(OH)D3(ng/ml), and 25(OH)D(ng/ml) were expressed as median (P25, P75).

Vitamin D data were not available for Dongfang and Baisha Li autonomous county due to an insufficient quantity of serum obtained in these regions.

^a^ 25(OH)D =25(OH)D2+25(OH)D3.

**Supplementary Table 3.** The region-specified prevalence of 25-hydroxyvitamin D deficiency across age groups **(China, 2022).**

| **Regions** | **0-3 y** | | |  | **4-6 y** | | |  | **7-12 y** | | |  | **13-18 y** | | |
| --- | --- | --- | --- | --- | --- | --- | --- | --- | --- | --- | --- | --- | --- | --- | --- |
|  | **Prevalence (%) (95% CI)** | **case** | **number** |  | **Prevalence (%) (95% CI)** | **case** | **number** |  | **Prevalence (%) (95% CI)** | **case** | **number** |  | **Prevalence (%) (95% CI)** | **case** | **number** |
| Haikou | 14.6 (11.5, 17.7) | 73 | 501 |  | 23.1 (21.2, 25.0) | 432 | 1870 |  | 28.2 (27.0, 29.5) | 1426 | 5050 |  | 42.9 (40.9, 44.9) | 995 | 2320 |
| Ding'an County | 1.0 (0.4, 3.0) | 3 | 288 |  | 6.0 (2.2, 9.8) | 9 | 150 |  | 24.4 (11.9, 37.0) | 11 | 45 |  | 37.7 (26.8, 48.5) | 29 | 77 |
| Danzhou | 3.5 (1.2, 5.7) | 9 | 258 |  | 10.2 (6.1, 14.4) | 21 | 205 |  | 46.8 (35.6, 57.9) | 36 | 77 |  | 59.6 (53.4, 65.8) | 143 | 240 |
| Lingao County | 1.6 (0.3, 2.8) | 6 | 381 |  | 7.5 (3.4, 11.5) | 12 | 161 |  | - | - | - |  | - | - | - |
| Chengmai County | 3.3 (0.1, 6.5) | 4 | 121 |  | 6.0 (1.3, 10.7) | 6 | 100 |  | 28.2 (17.7, 38.6) | 20 | 71 |  | 64.5 (56.6, 72.4) | 91 | 141 |
| Tunchang County | 0.6 (0.1, 3.1) | 1 | 180 |  | 1.4 (0.3, 7.7) | 1 | 70 |  | 4.8 (0.9, 22.7) | 1 | 21 |  | - | - | - |
| Wenchang | 1.1 (0.0, 2.2) | 4 | 359 |  | 4.9 (1.9, 7.9) | 10 | 204 |  | 43.1 (30.4, 55.8) | 25 | 58 |  | 34.3 (25.0, 43.7) | 34 | 99 |
| Qionghai | 3.9 (2.1, 5.7) | 17 | 441 |  | 3.6 (1.3, 5.9) | 9 | 251 |  | 42.3 (31.3, 53.3) | 33 | 78 |  | 38.3 (31.9, 44.7) | 85 | 222 |
| Wanning | 0.8 (0.1, 4.4) | 1 | 126 |  | 4.2 (1.7, 6.8) | 10 | 236 |  | 22.4 (17.8, 26.9) | 72 | 322 |  | 45.2 (35.0, 55.3) | 42 | 93 |
| Changjiang Li-AC | 1.0 (0.2, 5.4) | 1 | 101 |  | 5.1 (0.2, 10.0) | 4 | 78 |  | 12.5 (2.2, 47.1) | 1 | 8 |  | 37.3 (29.4, 45.3) | 53 | 142 |
| Dongfang | 4.0 (0.6, 7.4) | 5 | 126 |  | 11.6 (6.8, 16.4) | 20 | 172 |  | - | - | - |  | - | - | - |
| Sanya | 3.1 (1.3, 4.9) | 11 | 353 |  | 8.6 (5.7, 11.6) | 30 | 347 |  | 24.2 (20.9, 27.5) | 160 | 661 |  | 40.0 (33.5, 46.5) | 86 | 215 |
| Lingshui Li -AC | 3.7 (1.9, 5.6) | 15 | 402 |  | 5.6 (3.1, 8.1) | 18 | 323 |  | 53.8 (34.7, 73.0) | 14 | 26 |  | 50.6 (44.6, 56.5) | 137 | 271 |
| Ledong Li-AC | 1.9 (0.2, 3.5) | 5 | 264 |  | 5.5 (2.5, 8.5) | 12 | 220 |  | 10.2 (7.7, 12.6) | 58 | 571 |  | 32.5 (25.4, 39.7) | 54 | 166 |
| Baoting Li and Miao-AC | 4.1 (0.6, 7.6) | 5 | 122 |  | 5.1 (1.7, 8.5) | 8 | 157 |  | 15.8 (12.9, 18.6) | 98 | 622 |  | 40.5 (32.6, 48.5) | 60 | 148 |
| Wuzhishan | 1.0 (0.2, 5.5) | 1 | 99 |  | 2.5 (0.7, 8.8) | 2 | 79 |  | 25.7 (20.4, 30.9) | 68 | 265 |  | 66.0 (56.4, 75.5) | 62 | 94 |
| Qiongzhong Li and Miao-AC | 1.7 (0.5, 6.0) | 2 | 118 |  | 20.4 (12.8, 28.0) | 22 | 108 |  | 42.9 (37.1, 48.7) | 121 | 282 |  | 35.3 (28.1, 42.5) | 60 | 170 |
| Baisha Li-AC | - | - | - |  | - | - | - |  | - | - | - |  | 31.3 (22.8, 39.8) | 36 | 115 |

**Abbreviations:** AC, autonomous county.

**Note**: The VDD prevalence in Dongfang and Baisha Li-autonomous county in several age groups were not available due to the limited serum samples

**Supplementary Table 4**. Stratified levels of 25-hydroxyvitamin D2 and 25-hydroxyvitamin D3 in children and adolescence across age groups **(China, 2022).**

| **Covariates** | **0-3 y** | | | | **4-6 y** | | | | | **7-12 y** | | | | | **13-18 y** | | | | |
| --- | --- | --- | --- | --- | --- | --- | --- | --- | --- | --- | --- | --- | --- | --- | --- | --- | --- | --- | --- |
|  | **25(OH)D2** | ***p*** | **25(OH)D3** | ***p*** |  | **25(OH)D2** | ***p*** | **25(OH)D3** | ***p*** |  | **25(OH)D2** | ***p*** | **25(OH)D3** | ***p*** |  | **25(OH)D2** | ***p*** | **25(OH)D3** | ***p*** |
| **Overall** | 0.46 (0.27, 0.68) |  | 35.71 (29.16, 43.48) |  |  | 0.44 (0.24, 0.66) |  | 26.00 (22.12, 30.36) |  |  | 0.43 (0.23, 0.62) |  | 23.00 (19.36, 27.09) |  |  | 0.39 (0.22, 0.58) |  | 20.44 (16.79, 24.10) |  |
| **Gender** |  | 0.059 |  | **<0.001** |  |  | 0.394 |  | **<0.001** |  |  | 0.155 |  | **<0.001** |  |  | **<0.001** |  | **<0.001** |
| Male | 0.47 (0.28, 0.67) |  | 36.28 (29.99, 44.25) |  |  | 0.45 (0.24, 0.67) |  | 26.68 (22.70, 31.17) |  |  | 0.42 (0.22, 0.62) |  | 24.14 (20.52, 28.18) |  |  | 0.38 (0.21, 0.56) |  | 21.46 (17.93, 25.41) |  |
| Female | 0.45 (0.26, 0.71) |  | 34.93 (28.05, 42.60) |  |  | 0.43 (0.24, 0.65) |  | 25.41 (21.62, 29.50) |  |  | 0.43 (0.24, 0.63) |  | 21.89 (18.33, 25.87) |  |  | 0.41 (0.23, 0.62) |  | 19.47 (15.83, 22.92) |  |
| **Nationalities** |  | 0.324 |  | **<0.001** |  |  | 0.118 |  | **<0.001** |  |  | **0.001** |  | 0.993 |  |  | **0.047** |  | 0.131 |
| Han | 0.46 (0.27, 0.68) |  | 35.85 (29.07, 43.86) |  |  | 0.45 (0.24, 0.67) |  | 26.01 (22.02, 30.39) |  |  | 0.44 (0.23, 0.64) |  | 23.01 (19.46, 27.17) |  |  | 0.45 (0.25, 0.62) |  | 20.58 (16.79, 24.10) |  |
| Li | 0.47 (0.27, 0.78) |  | 36.45 (30.54, 43.88) |  |  | 0.41 (0.22, 0.66) |  | 26.95 (23.65, 31.58) |  |  | 0.46 (0.29, 0.69) |  | 23.30 (19.32, 27.21) |  |  | 0.40 (0.23, 0.58) |  | 20.76 (17.28, 24.82) |  |
| Others | 0.38 (0.22, 0.70) |  | 35.02 (28.72, 43.97) |  |  | 0.48 (0.29, 0.81) |  | 25.83 (22.63, 30.97) |  |  | 0.40 (0.22, 0.57) |  | 23.15 (18.99, 27.90) |  |  | 0.37 (0.23, 0.52) |  | 21.24 (17.92, 24.93) |  |
| **Regions** |  | 0.148 |  | **<0.001** |  |  | **0.008** |  | **<0.001** |  |  | **<0.001** |  | **<0.001** |  |  | **<0.001** |  | **<0.001** |
| Urban | 0.53 (0.31, 0.71) |  | 33.45 (27.07, 41.41) |  |  | 0.46 (0.26, 0.68) |  | 24.70 (20.70, 28.88) |  |  | 0.41 (0.21, 0.61) |  | 22.63 (18.97, 26.63) |  |  | 0.40 (0.22, 0.61) |  | 20.65 (16.83, 24.40) |  |
| Rural | 0.41 (0.23, 0.65) |  | 36.88 (30.46, 44.54) |  |  | 0.43 (0.22, 0.66) |  | 27.49 (23.94, 31.89) |  |  | 0.46 (0.30, 0.65) |  | 24.24 (20.54, 28.48) |  |  | 0.36 (0.22, 0.52) |  | 19.69 (16.52, 23.13) |  |
| **Quartile of BMI (kg/m2)** |  | **<0.001** |  | **<0.001** |  |  | 0.825 |  | **0.015** |  |  | **0.004** |  | **<0.001** |  |  | 0.709 |  | **0.038** |
| Q1 | 0.47 (0.27, 0.80) |  | 30.46 (25.88, 36.09) |  |  | 0.46 (0.26, 0.71) |  | 26.64 (22.64, 30.54) |  |  | 0.44 (0.26, 0.66) |  | 24.31 (20.79, 28.18) |  |  | 0.31 (0.18, 0.51) |  | 20.19 (18.34, 25.80) |  |
| Q2 | 0.48 (0.32, 0.71) |  | 31.08 (25.69, 36.98) |  |  | 0.46 (0.26, 0.72) |  | 25.94 (22.21, 30.21) |  |  | 0.44 (0.21, 0.65) |  | 23.33 (19.52, 27.56) |  |  | 0.46 (0.27, 0.64) |  | 21.05 (15.99, 23.82) |  |
| Q3 | 0.53 (0.32, 0.66) |  | 32.21 (26.33, 39.86) |  |  | 0.45 (0.25, 0.71) |  | 24.60 (21.58, 28.88) |  |  | 0.43 (0.24, 0.62) |  | 22.23 (18.90, 26.18) |  |  | 0.42 (0.24, 0.60) |  | 20.28 (16.78, 23.70) |  |
| Q4 | 0.53 (0.22, 0.75) |  | 32.89 (24.38, 39.58) |  |  | 0.38 (0.20, 0.63) |  | 24.84 (20.56, 30.36) |  |  | 0.44 (0.23, 0.65) |  | 22.39 (18.77, 25.81) |  |  | 0.44 (0.25, 0.62) |  | 20.91 (17.37, 24.56) |  |
| **Vitamin D supplement during gestation** |  | **0.017** |  | **<0.001** |  |  | **0.047** |  | **<0.001** |  |  | 0.133 |  | 0.864 |  |  | 0.171 |  | 0.252 |
| Never | 0.46 (0.28, 0.68) |  | 31.08 (26.24, 37.42) |  |  | 0.45 (0.25, 0.69) |  | 26.26 (22.60, 30.61) |  |  | 0.43 (0.23, 0.62) |  | 22.96 (19.35, 26.91) |  |  | 0.43 (0.24, 0.61) |  | 20.69 (16.81, 23.95) |  |
| Seldom | 0.54 (0.34, 0.78) |  | 27.94 (23.73, 32.33) |  |  | 0.47 (0.26, 0.73) |  | 24.98 (21.60, 29.19) |  |  | 0.45 (0.25, 0.67) |  | 23.24 (19.63, 27.18) |  |  | 0.45 (0.26, 0.62) |  | 20.72 (17.21, 24.46) |  |
| Often | 0.59 (0.36, 0.76) |  | 33.17 (26.69, 38.39) |  |  | 0.41 (0.27, 0.75) |  | 26.40 (23.29, 30.47) |  |  | 0.43 (0.23, 0.65) |  | 23.37 (19.38, 27.40) |  |  | 0.43 (0.27, 0.64) |  | 20.96 (17.98, 24.16) |  |
| Always | 0.56 (0.37, 0.69) |  | 37.08 (30.17, 43.43) |  |  | 0.46 (0.23, 0.73) |  | 29.45 (27.32, 34.58) |  |  | 0.47 (0.24, 0.72) |  | 22.55 (18.70, 27.39) |  |  | 0.34 (0.18, 0.50) |  | 21.22 (17.43, 25.69) |  |
| **Premature birth ^a^** |  | 0.054 |  | **<0.001** |  |  | 0.410 |  | 0.074 |  |  | 0.696 |  | 0.750 |  |  | 0.049 |  | 0.262 |
| Yes | 0.43 (0.30, 0.66) |  | 35.51 (29.89, 41.45) |  |  | 0.43 (0.24, 0.62) |  | 26.20 (22.93, 31.38) |  |  | 0.42 (0.25, 0.60) |  | 22.99 (19.60, 27.66) |  |  | 0.40 (0.24, 0.58) |  | 20.01 (16.51, 23.81) |  |
| No | 0.46 (0.27, 0.69) |  | 36.17 (29.82, 44.34) |  |  | 0.44 (0.24, 0.67) |  | 26.09 (22.04, 30.40) |  |  | 0.43 (0.23, 0.63) |  | 23.12 (19.33, 27.35) |  |  | 0.44 (0.25, 0.62) |  | 20.55 (16.88, 24.13) |  |
| **Conception ways** |  | 0.607 |  | 0.418 |  |  | 0.639 |  | 0.775 |  |  | 0.296 |  | 0.934 |  |  | 0.969 |  | 0.577 |
| Natural pregnancy | 0.46 (0.27, 0.70) |  | 36.02 (29.50, 43.91) |  |  | 0.44 (0.24, 0.67) |  | 26.25 (22.27, 30.56) |  |  | 0.44 (0.24, 0.64) |  | 23.08 (19.42, 27.18) |  |  | 0.44 (0.25, 0.61) |  | 20.64 (16.96, 24.28) |  |
| Assisted reproduction | 0.40 (0.27, 0.54) |  | 33.40 (27.37, 40.53) |  |  | 0.43 (0.22, 0.64) |  | 26.20 (21.97, 29.85) |  |  | 0.46 (0.26, 0.70) |  | 23.03 (18.99, 28.25) |  |  | 0.47 (0.24, 0.60) |  | 20.60 (15.10, 24.27) |  |
| **Delivery methods** |  | 0.461 |  | **<0.001** |  |  | 0.517 |  | 0.126 |  |  | 0.231 |  | 0.146 |  |  | 0.075 |  | 0.440 |
| Natural Childbirth | 0.46 (0.27, 0.68) |  | 36.03 (29.46, 44.17) |  |  | 0.44 (0.23, 0.67) |  | 26.33 (22.43, 30.57) |  |  | 0.44 (0.25, 0.64) |  | 23.02 (19.40, 27.09) |  |  | 0.44 (0.24, 0.61) |  | 20.70 (17.03, 24.32) |  |
| Cesarean section | 0.45 (0.27, 0.70) |  | 35.85 (29.41, 43.22) |  |  | 0.45 (0.25, 0.67) |  | 25.92 (21.89, 30.43) |  |  | 0.43 (0.22, 0.64) |  | 23.31 (19.64, 27.50) |  |  | 0.46 (0.26, 0.65) |  | 20.44 (16.41, 24.42) |  |
| **Number of births** |  | 0.176 |  | **<0.001** |  |  | 0.331 |  | 0.712 |  |  | 0.302 |  | 0.748 |  |  | 0.256 |  | 0.871 |
| Single birth | 0.46 (0.28, 0.70) |  | 36.14 (29.82, 44.16) |  |  | 0.44 (0.24, 0.67) |  | 26.18 (22.24, 30.53) |  |  | 0.44 (0.24, 0.64) |  | 23.10 (19.44, 27.19) |  |  | 0.44 (0.25, 0.62) |  | 20.63 (16.98, 24.30) |  |
| Multiple births | 0.37 (0.21, 0.56) |  | 31.89 (25.37, 38.14) |  |  | 0.43 (0.21, 0.66) |  | 26.54 (22.60, 30.62) |  |  | 0.46 (0.29, 0.65) |  | 22.85 (19.10, 27.11) |  |  | 0.42 (0.23, 0.56) |  | 21.06 (15.85, 24.07) |  |
| **Birth weight (kg)** |  | 0.521 |  | **<0.001** |  |  | 0.978 |  | **<0.001** |  |  | 0.587 |  | 0.588 |  |  | 0.763 |  | 0.853 |
| <2.5 | 0.39 (0.21, 0.67) |  | 37.69 (31.50, 44.95) |  |  | 0.45 (0.23, 0.74) |  | 27.24 (23.75, 32.48) |  |  | 0.46 (0.28, 0.65) |  | 23.28 (20.05, 27.95) |  |  | 0.41 (0.24, 0.58) |  | 20.69 (17.25, 24.65) |  |
| 2.5-4.0 | 0.47 (0.28, 0.69) |  | 36.41 (29.97, 44.32) |  |  | 0.44 (0.24, 0.67) |  | 25.97 (22.02, 30.37) |  |  | 0.44 (0.24, 0.65) |  | 23.20 (19.40, 27.23) |  |  | 0.44 (0.24, 0.62) |  | 20.75 (17.01, 24.15) |  |
| >4.0 | 0.41 (0.24, 0.63) |  | 30.52 (25.51, 37.32) |  |  | 0.44 (0.24, 0.66) |  | 26.87 (23.36, 31.51) |  |  | 0.44 (0.24, 0.64) |  | 23.03 (19.35, 27.23) |  |  | 0.43 (0.26, 0.62) |  | 20.47 (16.60, 24.41) |  |
| **Exclusively breastfed for 6 months or more** |  | 0.589 |  | **<0.001** |  |  | 0.922 |  | **0.001** |  |  | 0.092 |  | 0.939 |  |  | 0.686 |  | 0.992 |
| Yes | 0.45 (0.27, 0.68) |  | 33.94 (27.75, 41.79) |  |  | 0.44 (0.24, 0.67) |  | 26.01 (22.15, 30.37) |  |  | 0.44 (0.24, 0.65) |  | 23.09 (19.37, 27.17) |  |  | 0.44 (0.24, 0.62) |  | 20.65 (16.96, 24.31) |  |
| No | 0.47 (0.28, 0.70) |  | 38.28 (32.04, 46.12) |  |  | 0.45 (0.24, 0.67) |  | 26.72 (22.77, 31.54) |  |  | 0.42 (0.22, 0.63) |  | 23.00 (19.56, 27.19) |  |  | 0.44 (0.28, 0.61) |  | 20.60 (16.90, 23.98) |  |
| **Growth and development assessment** |  | **0.037** |  | **<0.001** |  |  | **0.026** |  | **<0.001** |  |  | 0.479 |  | **<0.001** |  |  | 0.389 |  | 0.224 |
| Retardation | 0.53 (0.28, 0.82) |  | 37.15 (31.10, 44.68) |  |  | 0.46 (0.23, 0.73) |  | 26.54 (22.86, 30.69) |  |  | 0.43 (0.25, 0.63) |  | 24.53 (20.79, 28.41) |  |  | 0.37 (0.20, 0.55) |  | 20.21 (15.60, 24.42) |  |
| Normal | 0.45 (0.27, 0.67) |  | 36.55 (30.10, 44.52) |  |  | 0.44 (0.24, 0.67) |  | 25.82 (21.93, 30.16) |  |  | 0.42 (0.23, 0.62) |  | 22.83 (19.24, 26.89) |  |  | 0.40 (0.22, 0.59) |  | 20.54 (16.90, 24.15) |  |
| Obesity | 0.49 (0.32, 0.68) |  | 36.28 (29.59, 46.07) |  |  | 0.38 (0.21, 0.54) |  | 23.75 (20.18, 28.19) |  |  | 0.42 (0.23, 0.63) |  | 22.67 (18.59, 26.73) |  |  | 0.36 (0.20, 0.60) |  | 20.14 (16.66, 23.44) |  |
| **Family annual income (yuan)** |  | 0.105 |  | **<0.001** |  |  | 0.061 |  | **<0.001** |  |  | **0.030** |  | 0.285 |  |  | 0.111 |  | 0.596 |
| 0-50,000 | 0.45 (0.26, 0.68) |  | 36.91 (30.37, 44.79) |  |  | 0.45 (0.24, 0.67) |  | 26.60 (22.90, 30.94) |  |  | 0.45 (0.25, 0.65) |  | 23.12 (19.50, 27.25) |  |  | 0.43 (0.24, 0.61) |  | 20.61 (16.90, 24.27) |  |
| 50,000-100,000 | 0.49 (0.29, 0.69) |  | 35.03 (29.20, 42.33) |  |  | 0.47 (0.26, 0.71) |  | 25.31 (21.33, 29.80) |  |  | 0.42 (0.22, 0.63) |  | 23.14 (19.50, 27.02) |  |  | 0.47 (0.26, 0.64) |  | 20.76 (17.50, 24.29) |  |
| ＞100,000 | 0.46 (0.28, 0.72) |  | 31.20 (25.67, 40.02) |  |  | 0.41 (0.24, 0.64) |  | 25.09 (20.69, 29.48) |  |  | 0.41 (0.21, 0.63) |  | 22.80 (18.89, 26.89) |  |  | 0.44 (0.26, 0.61) |  | 20.48 (16.54, 24.42) |  |

**Supplementary Table 5**. Climate-specific zones stratified levels of serum 25-hydroxyvitamin D2 and 25-hydroxyvitamin D3 across age groups **(China, 2022).**

| **Climate-specific zones** | **0-3 y** | | | |  | **4-6 y** | | | |  | **7-12 y** | | | |  | **13-18 y** | | | | |
| --- | --- | --- | --- | --- | --- | --- | --- | --- | --- | --- | --- | --- | --- | --- | --- | --- | --- | --- | --- | --- |
|  | **25(OH)D2** | ***p*** | **25(OH)D3** | ***p*** |  | **25(OH)D2** | ***p*** | **25(OH)D3** | ***p*** |  | **25(OH)D2** | ***p*** | **25(OH)D3** | ***p*** |  | | **25(OH)D2** | ***p*** | **25(OH)D3** | ***p*** |
|  |  | **<0.001** |  | **<0.001** |  |  | **<0.001** |  | **<0.001** |  |  | **<0.001** |  | **<0.001** |  | |  | **<0.001** |  | **<0.001** |
| **Humid zone** | 0.38 (0.24, 0.54) |  | 30.68 (25.88, 36.70) |  |  | 0.40 (0.22, 0.61) |  | 28.69 (24.83, 33.27) |  |  | 0.39 (0.25, 0.53) |  | 23.10 (19.33, 27.47) |  |  | | 0.43 (0.27, 0.58) |  | 21.05 (18.08, 24.40) |  |
| **Sub-humid zone** | 0.52 (0.29, 0.70) |  | 35.30 (28.00, 44.31) |  |  | 0.44 (0.23, 0.67) |  | 24.98 (20.83, 29.32) |  |  | 0.40 (0.20, 0.59) |  | 22.66 (18.99, 26.72) |  |  | | 0.38 (0.20, 0.59) |  | 20.24 (16.35, 23.59) |  |
| **Mountainous humid zone** | 0.56 (0.38, 0.99) |  | 37.84 (30.83, 45.54) |  |  | 0.53 (0.32, 0.88) |  | 25.42 (22.43, 29.10) |  |  | 0.42 (0.27, 0.64) |  | 21.86 (18.17, 25.89) |  |  | | 0.36 (0.23, 0.63) |  | 21.61 (17.33, 25.90) |  |
| **Semi-arid and sub-humid zone** | 0.42 (0.22, 0.67) |  | 35.64 (29.64, 43.99) |  |  | 0.46 (0.27, 0.67) |  | 26.70 (23.41, 30.61) |  |  | 0.51 (0.36, 0.70) |  | 24.18 (20.75, 28.35) |  |  | | 0.40 (0.24, 0.57) |  | 20.66 (17.05, 24.99) |  |
| **Semi-arid zone** | 0.47 (0.21, 0.69) |  | 31.63 (27.13, 39.53) |  |  | 0.41 (0.19, 0.66) |  | 26.73 (23.50, 30.88) |  |  | 0.46 (0.20, 0.76) |  | 29.26 (23.10, 31.60) |  |  | | 0.46 (0.37, 0.62) |  | 20.74 (17.70, 24.13) |  |

**Note:** Levels of 25(OH)D2(ng/ml) and 25(OH)D3(ng/ml) were expressed as median (P25, P75).

**Supplementary Table 6.** The multivariable logistic regression analysis of vitamin D deficiency **(China, 2022).**

| **Covariates** | **Ref** | ***β*** | ***SE*** | ***Wald*** | ***OR*（95%*CI*）** | ***P*** |
| --- | --- | --- | --- | --- | --- | --- |
| **Age** |  | 0.175 | 0.008 | 535.758 | 1.192 (1.174-1.210) | **<0.001** |
| **Gender** |  |  |  |  |  |  |
| Female | Male | 0.537 | 0.052 | 104.572 | 1.710 (1.543-1.896) | **<0.001** |
| **Nationalities** |  |  |  |  |  |  |
| Li | Han | -0.277 | 0.085 | 10.769 | 0.758 (0.642-0.894) | **0.001** |
| **Regions** |  |  |  |  |  |  |
| Rural | Urban | -0.532 | 0.068 | 61.507 | 0.587 (0.514-0.671) | **<0.001** |
| **Exclusively breastfed for 6 months or more** |  |  |  |  |  |  |
| Yes | No | 0.357 | 0.077 | 21.419 | 1.429 (1.228-1.662) | **<0.001** |
| **Growth and development assessment** |  |  |  |  |  |  |
| Normal | Retardation | 0.323 | 0.106 | 9.283 | 1.381 (1.122-1.700) | **0.002** |
| Obesity |  | 0.586 | 0.171 | 11.724 | 1.796 (1.285-2.512) | **0.001** |
| **Family annual income (yuan)** |  |  |  |  |  |  |
| ＞100,000 | 0-50,000 | 0.363 | 0.074 | 24.096 | 1.438 (1.244-1.662) |  |

**Supplementary Table 7. Season stratified prevalence of 25-hydroxyvitamin D deficiency across age groups (China, 2022).**

| **Season** | **0-3 y** | | | |  | **4-6 y** | | |  | **7-12 y** | | |  | **13-18 y** | | |
| --- | --- | --- | --- | --- | --- | --- | --- | --- | --- | --- | --- | --- | --- | --- | --- | --- |
|  | **Prevalence (%) (95% CI)** | | **case** | **Testing sample** |  | **Prevalence (%) (95% CI)** | **case** | **Testing sample** |  | **Prevalence (%) (95% CI)** | **case** | **Testing sample** |  | **Prevalence (%) (95% CI)** | **case** | **Testing sample** |
| **Spring** | | 1.8 (1.2, 2.5) | 30 | 1635 |  | 5.8 (4.0, 7.5) | 40 | 694 |  | 11.1 (1.9, 43.5) | 1 | 9 |  | 0.0 (0.0, 0.0) | 0 | 2 |
| **Summer** | | 5.9 (4.1, 7.6) | 41 | 699 |  | 5.9 (4.6, 7.1) | 84 | 1432 |  | 0.0 (0.0, 0.0) | 0 | 13 |  | 0.0 (0.0, 0.0) | 0 | 3 |
| **Autumn** | | 14.1 (11.0, 17.1) | 70 | 497 |  | 22.9 (21.0, 24.9) | 406 | 1772 |  | 28.9 (27.5, 30.2) | 1255 | 4348 |  | 43.0 (40.9, 45.1) | 923 | 2148 |
| **Winter** | | 1.1 (0.5, 1.6) | 16 | 1505 |  | 11.5 (9.4, 13.7) | 96 | 833 |  | 23.4 (22.0, 24.7) | 888 | 3800 |  | 44.2 (42.2, 46.2) | 1043 | 2360 |

**Note**: Spring:12-2 month; Summer:3-5 month; Autumn:6-8 month; Winter:9-12 month
